# Exposure to risperidone versus other antipsychotics and risk of osteoporosis‐related fractures: a population‐based study

**DOI:** 10.1111/acps.13101

**Published:** 2019-10-11

**Authors:** E. Clapham, R. Bodén, J. Reutfors, T. Svensson, D. Ramcharran, H. Qiu, H. Kieler, S. Bahmanyar

**Affiliations:** ^1^ Centre for Pharmacoepidemiology (CPE) Department of Medicine Solna Karolinska University Hospital Karolinska Institutet Solna Sweden; ^2^ Department of Neuroscience, Psychiatry Uppsala University Uppsala Sweden; ^3^ Janssen Global Research and Development Epidemiology Titusville NJ USA

**Keywords:** risperidone, atypical antipsychotics, osteoporosis, fracture

## Abstract

**Objective:**

Antipsychotics may increase serum prolactin, which has particularly been observed with risperidone. Further, hyperprolactinemia has been linked to osteoporosis‐related fractures. Therefore, we investigated fracture risk in a nationwide cohort exposed to antipsychotics.

**Methods:**

Swedish registers were used to identify adults with two consecutive dispensations of risperidone (*n* = 38 211), other atypical antipsychotics not including paliperidone (*n* = 60 691), or typical antipsychotics (*n* = 17 445) within three months between 2006 and 2013. An osteoporosis‐related fracture was defined as a non‐open hip/femur fracture in primary analyses. Cox regression was used to estimate hazard ratios (HRs) and 95% confidence intervals (CIs).

**Results:**

Risperidone users were on average older (mean age of 68, 44, and 63 years for risperidone, other atypical antipsychotics, and typical antipsychotics respectively). Compared with other atypical antipsychotics, there was no association between risperidone and osteoporosis‐related fractures in the overall (HR = 1.04, CI: 0.91–1.19) or age‐stratified analyses. A significantly increased risk of typical antipsychotics (HR = 1.24, CI: 1.07–1.45) compared with other atypical antipsychotics remained for ages >45 years.

**Conclusion:**

Risperidone does not appear to be associated with an increased risk of osteoporosis‐related fracture compared with other atypical antipsychotic agents as a group. For typical antipsychotics, a moderately elevated risk of hip fractures was noted compared with other atypical antipsychotics, possibly because of residual confounding.

**Significant Outcomes**

- Exposure to risperidone, an antipsychotic with serum prolactin‐elevating properties, was not associated with an increased risk of fractures commonly related to osteoporosis compared with other atypical antipsychotics.
- Compared with other atypical antipsychotics, exposure to typical antipsychotics was associated with a moderately increased risk of osteoporosis‐related fractures.

**Limitations**

- Confounding can never be fully accounted for in an observational study.
- Although the prescribed drug register contains information on filled prescription, it is not known whether the patient actually ingested the medication as prescribed.

## Introduction

It is well known that the use of antipsychotic agents can cause elevated levels of circulating prolactin, because of their antidopaminergic activity affecting the tuberoinfundibular pathway 1. Elevated prolactin levels are associated with decreased bone mineral density 2. In addition, some epidemiological studies suggest a possible link between an elevated prolactin level and an increased risk of osteoporosis‐related fractures 1, 3. Risperidone is an atypical antipsychotic agent featuring antagonistic effects of the dopamine type 2 and serotonin type 2A receptors. It is associated with a greater and more frequent elevation of circulating prolactin level compared with other atypical antipsychotics 4, 5, and because of this fact, there has been a concern that risperidone may be associated with an increased risk of osteoporosis‐related fractures.

The risk of fractures might also be elevated among users of antipsychotics for reasons other than osteoporosis induced by hyperprolactinemia. For example, other side‐effects of antipsychotics, as well as comorbid somatic illnesses, could contribute to fractures. In early 2017, the U.S. Food and Drug Agency approved a labeling update for all antipsychotic medications stating that antipsychotic drugs may cause somnolence, postural hypotension, and motor and sensory instability, which may lead to falls and, consequently, fractures or other injuries 6.

The importance of antipsychotic‐induced hyperprolactinemia in bone mineral loss remains undetermined 7. While the use of antipsychotics has been associated with increased risk of fracture in some epidemiological studies 8, 9, 10, the precise role of prolactin‐raising drugs compared with other factors contributing to osteoporosis and fracture has not been disentangled 11.

### Aims of the study

The goal of this nationwide cohort study was to compare the risk of osteoporosis‐related fractures associated with risperidone, other atypical antipsychotics, and typical antipsychotics. Using an empirical definition of osteoporosis‐related fracture, we aimed to evaluate the incidence of both hip and non‐hip fractures, which were defined as primary and secondary outcomes respectively.

## Material and methods

### Data sources

Data were obtained from five different Swedish national registers, which were linked using the unique personal identification number (PIN), which is assigned to all Swedish residents at birth or immigration 12. The Prescribed Drug Register (PDR) provided information on antipsychotic exposure, classified as risperidone, any other atypical antipsychotics (except for paliperidone, the active metabolite of risperidone), or typical antipsychotics using the Anatomical Therapeutic Chemical (ATC) classification codes. All formulations of new users of antipsychotics were included (i.e., oral and injectable). The register contains information on all dispensed drugs at Swedish pharmacies since July 2005 13 and includes information on ATC classification, quantity, and dates of dispensing. The medical indication of the drug is not included in the register. The PDR does not include drugs administered during hospitalization.

The National Patient Register (NPR) provides information on fractures and some potential confounding factors (see ‘variables’ section). The register contains details of in‐patient discharge diagnoses, which have been recorded since 1964 with complete national coverage from 1987 14. Since 2001, information on out‐patient visits to hospital (specialist care) is registered and the coverage improved during the following years. Diagnoses are coded according to the International Classification of Disease (ICD) version 10 since 1997. The quality of the register has been shown to be of a high standard 14.

The Cause of Death Register 15, Swedish Cancer Register 16, and Register of Population and Population Changes provided information on important covariates and censoring variables (see ‘variables’ section).

### Study population and design

We performed a cohort study using data from national longitudinal population‐based registers in Sweden. The study included a cohort of patients with different antipsychotic exposures: risperidone, any other atypical antipsychotics (except for paliperidone, the active metabolite of risperidone), or typical antipsychotics. Patients were excluded if they had active cancer or a pituitary tumor according to the Swedish Cancer Register (at index date or within 5 years prior), or had at any time prior to the start of follow‐up received a dispensing of paliperidone (ATC code N05AX13). Further, patients with a non‐open hip/femur fracture—commonly related to osteoporosis 17—prior to the exposure index date, or within 6 months after the exposure index date, were excluded from the study population.

Eligible study subjects were patients, at least 18 years of age at index date, who were residents of Sweden at least 12 months prior to the first exposure of at least two consecutive dispensings of an antipsychotic between July 1, 2006, and December 31, 2014, and did not emigrate from Sweden for at least 12 months after the exposure index date. Subjects were followed longitudinally for the ascertainment of any new occurrences of osteoporosis‐related fractures until December 31, 2014.

Exposed individuals had to be new users of the antipsychotic, defined as having no prescription record for the same antipsychotic in the 12 months prior to the first dispensation that defined the exposure group. Subjects had to have two consecutive dispensings of an antipsychotic, with the second dispensing defined as the exposure index date. Consecutive dispensing was defined as one dispensing after another within 3 months and no other antipsychotics between the two dispensings. The 3‐month cutoff was pragmatically chosen because ongoing drug treatments in Sweden typically are prescribed three months at a time and we wanted to exclude the possibility of those who collected the prescription once and probably did not use it. There were three antipsychotic exposure cohorts: (i) risperidone, (ii) other atypical antipsychotics, and (iii) typical antipsychotics. Those with no previous prescription record for any antipsychotic in the 12 months prior to the first identified prescription during the study period were considered as treatment‐naïve individuals.

Cases of newly diagnosed fractures that met our empirical definition of being osteoporosis‐related (see section ‘variables’ below) were identified by the presence of a diagnosis in the NPR coded according to ICD. The primary outcome was non‐open hip/femur fractures. An in‐patient diagnosis was required for defining hip/femur fracture. Osteoporosis‐related fractures were empirically defined as non‐open fractures that occur in the absence of major traumas or bone metastases. Non‐hip/femur fractures were the secondary outcome and defined as fracture at the spine, rib, clavicle, humerus, radius/ulna, wrist, pelvis, or tibia/fibula. Non‐hip/femur fractures were identified from both in‐patient (including emergency room) and out‐patient diagnoses recorded in the NPR.

As early occurrence of fracture is not likely related to the exposure, cohort follow‐up began from 6 months after the exposure index date. The main analysis followed a time on drug approach. The active treatment follow‐up time ended at discontinuation of the treatment regimen plus 6 months, occurrence of an osteoporosis‐related fracture, emigration, or end of study period (December 31, 2014), whichever occurred earlier. Individuals were censored if they switched to an antipsychotic from another treatment group.

### Variables

To adjust for potential confounding factors, the prevalence of comorbidities such as psychiatric conditions and somatic diseases associated with elevated levels of prolactin was used based on previously recorded diagnoses in the NPR. Further, data on medication treatments for hyperprolactinemia and drugs other than antipsychotic medication associated with increased prolactin levels were collected from the PDR. Any previous psychiatric diagnoses recorded prior to the first of the two consecutive dispensations were considered as potential confounding factors, whereas information on previous somatic conditions and medication was collected starting 6 months prior to the exposure index date. Additional factors associated with the risk of osteoporosis were identified based on the FRAX fracture risk assessment tool 18. These potential confounding factors were obesity, earlier fracture during adulthood, risk factors for secondary osteoporosis, rheumatoid arthritis, nicotine use disorder, disease related to alcohol use disorder and treatment with cortisone, lithium, antiepileptics, or osteoporosis‐related medications. ICD and ATC codes of the investigated potential confounding factors as well as diagnostic codes for the primary and secondary outcomes are given in section 1 and section 3 of the Supporting information.

### Statistical analyses

Fracture incidence rates were estimated for each of the exposure groups. Analyses were conducted both for the outcomes of non‐open hip/femur and non‐hip/femur fractures. To exclude potential prevalent cases, and increased fracture risk resulting from previous injuries, an occurrence of a fracture was considered newly diagnosed, or incident, only if there was no ICD‐10 record of a fracture at the same site any time prior to exposure onset (side not considered). The fracture incidence rate was analyzed separately in treatment‐naïve individuals. The incidence rates of fractures were estimated according to the person‐time of the total cohort follow‐up, as well as the person‐time of active treatment exposure follow‐up by the three exposure groups (risperidone, other atypical antipsychotics, and typical antipsychotics). Some 20% of the patients who switched from one antipsychotic to another or treated with more than one antipsychotic drug were excluded.

Cox proportional hazards regression models were used to estimate unadjusted and adjusted hazard ratios (HRs and aHRs) and 95% confidence intervals (CIs) for fractures among the exposure groups. The reference group was other atypical antipsychotics, and main analyses were made using a time on drug approach. As there was substantial variation in the age distribution of the exposure groups, in addition to adjusting for age as a continuous variable, the analyses were also stratified by age groups (18–44, 45–64, and 65+ years). We also performed analyses based on the intention‐to‐treat method, where exposure time was not halted when the index antipsychotic ceased to be filled. Sensitivity analyses were conducted to check the robustness of the findings (see the Supporting information).

Covariates were retained in the final model if their inclusion, in a model containing the single covariate and the antipsychotic exposure variable, changed the HR for the antipsychotic exposure variable by 10% or more, relative to the unadjusted HR for antipsychotic exposure (i.e., adjusted HR/unadjusted HR is outside the interval 0.90–1.10), the so‐called ‘change‐in‐estimate’ criterion. The analysis of potential confounding factors was repeated when the cohort analysis was age‐stratified. The variables that fulfilled the 10% change‐in‐estimate criterion for the overall analysis were age, clinic of first dispensation, multidose dispensing, Charlson comorbidity index, history of psychiatric in‐patient care, dementia, and stress‐related or somatoform disorder. Multidose dispensation indicated that medication was delivered in separate bags or trays for each individual administration time, instead of separate pharmaceutical packaging for each drug (mainly used by elderly and certain individuals with psychiatric illness). Adjusting for antidepressant use slightly attenuated the risk of fracture, but the effect did not meet the required threshold for this measure to be retained as a covariate in regression models. The selection of confounding factors was repeated for each age stratum and did not reveal any new covariates which met the criteria to be included in the models for stratified analyses (data in section 4 of the Supporting information).

The assumption that hazard functions are proportional over time (i.e., constant relative hazard) was checked using a proportionality test and ‘log(‐log(survival)) vs. log of survival time’ plots. In addition, graphical assessment of time trend was conducted by ‘log(‐log(survival) vs. log of survival time’ plots, scaled Schoenfeld residual plot, and Kaplan–Meier survival curves.

Data management and analyses were conducted utilizing sas 9.4 software.

### Ethics

The study was approved by the regional ethical review board in Stockholm (ref nr 2016/541‐32).

## Results

The study included 38 211 individuals exposed to risperidone, 60 691 individuals exposed to other atypical antipsychotics, and 17 445 individuals exposed to typical antipsychotics (Table 1).

**Table 1:** Baseline characteristics of patients exposed to risperidone, other atypical antipsychotics, and typical antipsychotics

Characteristics	Risperidone		Other atypical antipsychotic*		Typical antipsychotic†	
	Number	Percent/SD	Number	Percent/SD	Number	Percent/SD
Total number included	38 211		60 691		17 445	
Sex, *N* (%)						
Male	15 672	41.0	28 120	46.3	7102	40.7
Female	22 539	59.0	32 571	53.7	10 343	59.3
Age at inclusion						
Mean (SD)	67.9	21.5	44.4	17.4	63.3	19.1
Age group, *N* (%)						
18–44	7202	18.8	32 579	53.7	3217	18.4
45–54	2919	7.6	11 456	18.9	2621	15.0
55–64	3144	8.2	8028	13.2	2928	16.8
65–74	4357	11.4	4642	7.6	2635	15.1
75+	20 589	53.9	3986	6.6	6044	34.6
History of psychiatric conditions						
Dementia	7065	18.5	1210	2.0	1670	9.6
Other organic psychiatric disorders	3690	9.7	2172	3.6	1257	7.2
Alcohol use disorder	2043	5.3	7659	12.6	1284	7.4
Other substance use disorders	1592	4.2	8033	13.2	1056	6.1
Schizophrenia	1057	2.8	2709	4.5	1176	6.7
Other psychosis	3061	8.0	6955	11.5	2013	11.5
Bipolar disorder	958	2.5	5635	9.3	514	2.9
Unipolar disorder	5760	15.1	19 184	31.6	2460	14.1
Other mood disorders	742	1.9	3270	5.4	419	2.4
Neurotic stress‐related or somatoform disorder	5169	13.5	20 130	33.2	2525	14.5
Personality disorder	1350	3.5	6195	10.2	884	5.1
Mental retardation and autism	1116	2.9	2012	3.3	401	2.3
Suicide attempt	1766	4.6	8099	13.3	940	5.4
Psychiatric in‐patient care within 180 days						
None	31 790	83.2	37 548	61.9	14 446	82.8
1–3	608	1.6	2433	4.0	301	1.7
4–21	2349	6.1	8992	14.8	1014	5.8
22+	3464	9.1	11 718	19.3	1684	9.7
Charlson comorbidity index						
Score 0	20 168	52.8	49 390	81.4	10 613	60.8
Score 1	9556	25	7156	11.8	3444	19.7
Score 2+	8487	22.2	4145	6.8	3388	19.4
Multidose dispensing						
Multidose drug dispensing‡	11 899	31.1	6978	11.5	3331	19.1
Package	26 312	68.9	53 713	88.5	14 114	80.9
Clinic of the prescriber of the index exposure						
No information	32	0.1	87	0.1	26	0.1
Primary care	17 772	46.5	3449	5.7	6660	38.2
Other	2806	7.3	2716	4.5	1225	7.0
Somatic clinic	4403	11.5	2490	4.1	1801	10.3
Psychiatric clinic	13 198	34.5	51 949	85.6	7733	44.3

* Atypical antipsychotics: aripiprazole (N05AX12), clozapine (N05AH02), lurasidone (N05AE05), olanzapine (N05AH03), quetiapine (N05AH04), risperidone (N05AX08), sertindole (N05AE03), and ziprasidone (N05AE04).

^†^ Typical antipsychotics (* indicates currently not in use in Sweden): chlorpromazine (N05AA01*), levomepromazine (N05AA02), promazine (N05AA03*), fluphenazine (N05AB02), perphenazine (N05AB03), prochlorperazine (N05AB04*), trifluoperazine (N05AB06*), thioridazine (N05AC02*), haloperidol (N05AD01), melperone (N05AD03), droperidol (N05AD08), molindone (N05AE02*), flupentixol (N05AF01), chlorprothixene (N05AF03), tiotixene (N05AF04*), zuclopenthixol (N05AF05), pimozide (N05AG02*), and loxapine (N05AH01).

^‡^ Multidose dispensing means that medication is delivered in separate bags or trays for each individual administration time, instead of separate pharmaceutical packaging for each drug (mainly used by elderly and some individuals with psychiatric illness).

The mean age ranged from 44 years (SD 17) among new users of atypical antipsychotics other than risperidone to 68 years (SD 21) among new users of risperidone. Most (53.9%) of new users of risperidone were 75 years or older at the time of the first dispensing, compared with only 6.6% among new users of other atypical antipsychotics.

Among risperidone users, 18.5% had a diagnosis of dementia, compared with 2.0% among users of other atypical antipsychotics (Table 1). A schizophrenia diagnosis occurred most frequently among user of typical antipsychotics (6.7%), while a unipolar or bipolar disorder was most common among users of other atypical antipsychotics (31.6% and 9.3% respectively). A history of suicide attempt was present among 13.3% of users of other atypical antipsychotics but only among 4.6% of risperidone users.

Psychiatric hospitalizations within 180 days prior to index exposure were most common in the other atypical antipsychotics group (38.1% of patients), and the corresponding percentages for the risperidone and typical groups were similar at about 17%. Clinic of the prescriber of the index antipsychotic was primary care for 46.5% risperidone users but 5.7% for users of other atypical antipsychotics.

Table 2 shows the association between the use of antipsychotic agents and the primary outcome of non‐open hip/femur fractures and applying a time on drug analysis, with associations reported as unadjusted and adjusted hazard ratios. In adjusted analyses, there was no statistically significant difference in the risk of fracture for risperidone compared with other atypical antipsychotics (aHR: 1.04; 95% CI: 0.91–1.19) and this was also the case in age‐stratified analyses. For typical antipsychotics, there was a 24% statistically significant higher risk (aHR: 1.24; 95% CI: 1.07–1.45), compared with other atypical antipsychotics. Following age stratification, the risk increase remained statistically significant for the age groups 45–64 years (aHR: 1.56; 95% CI: 1.05–2.32) and 65+ years (aHR: 1.21; 95% CI: 1.03–1.43). In the 18–44‐year age group, the number of fractures was eight, two, and two for risperidone, other atypical antipsychotics, and typical antipsychotics respectively. Corresponding analyses based on intention‐to‐treat yielded consistent results (see section 4 of the Supporting information).

**Table 2:** Primary outcome (non‐open hip/femur fractures). Hazard ratios (HRs) and 95% confidence intervals (CIs) for the association between the use of risperidone, other atypical antipsychotics, and typical antipsychotics and the primary outcome, overall and stratified by three age groups using a time on drug analysis

Characteristics	Person‐years of follow‐up	Number of events	Events per 100 000 person‐years	Unadjusted HR (95% CI)	Adjusted HR (95% CI)*
Other atypical antipsychotics	111 721	420	375.9	Reference	Reference
Risperidone	70 831	1269	1791.6	4.70 (4.21–5.25)	1.04 (0.91–1.19)
18–44†	13 926	2	14.4	0.85 (0.18–4.02)	0.99 (0.21–4.73)
45–64	14 797	41	277.1	1.44 (0.98–2.11)	1.03 (0.69–1.55)
65+	42 107	1226	2911.6	1.59 (1.41–1.80)	1.02 (0.88–1.17)
Typical antipsychotics	36 804	443	1203.7	3.17 (2.77–3.62)	1.24 (1.07–1.45)
18–44	6276	2	31.9	1.97 (0.42–9.31)	2.40 (0.49–11.81)
45–64	15 376	46	299.2	1.52 (1.05–2.20)	1.56 (1.05–2.32)
65+	15 153	395	2606.7	1.40 (1.21–1.62)	1.21 (1.03–1.43)

* Adjusted for age, clinic, multidose dispensing, Charlson index, history of psychiatric in‐patient care, dementia, and stress‐related or somatoform disorder.

^†^ Reference group for age‐stratified analysis is other atypical antipsychotics with similar age distribution.

Table 3 provides results for treatment‐naïve individuals. For risperidone compared with other atypical antipsychotics, there was no statistically significant difference in the risk of non‐open hip/femur fractures. For typical antipsychotics, there was a statistically significant risk increase compared with other atypical antipsychotics in the aggregated analysis (aHR: 1.25; 95% CI: 1.07–1.47) as well as in the age groups 45–64 years (aHR: 1.58; 95% CI: 1.01–2.50) and 65 + years (aHR: 1.23; 95% CI: 1.03–1.46).

**Table 3:** Primary outcome (non‐open hip/femur fractures), restricted to treatment‐naïve patients. Hazard ratios (HR) and 95% confidence intervals (CI) for the association between the use of risperidone, other atypical antipsychotics, and typical antipsychotics and the primary outcome, overall and stratified by three age groups using a time on drug analysis

Characteristics	Person‐years of follow‐up	Number of events	Events per 100 000 person‐years	Unadjusted HR (95% CI)	Adjusted HR (95% CI)*
Other atypical antipsychotics	89 405	342	382.5	Reference	Reference
Risperidone	61 774	1177	1905.3	4.90 (4.34–5.53)	1.04 (0.90–1.21)
18–44†	11 425	2	17.5	1.09 (0.22–5.43)	1.27 (0.25–6.54)
45–64	11 689	30	256.7	1.56 (0.99–2.47)	0.99 (0.60–1.63)
65+	38 660	1145	2961.7	1.57 (1.38–1.79)	1.02 (0.88–1.18)
Typical antipsychotics	34 725	426	1226.8	3.17 (2.75–3.66)	1.25 (1.07–1.47)
18–44	6025	2	33.2	2.19 (0.44–10.93)	2.78 (0.53–14.54)
45–64	14 266	40	280.4	1.67 (1.10–2.55)	1.58 (1.01–2.5)
65+	14 435	384	2660.2	1.40 (1.20–1.63)	1.23 (1.03–1.46)

* Adjusted for age, clinic, multidose dispensing, Charlson index, history of psychiatric in‐patient care, dementia, and stress‐related or somatoform disorder.

^†^ Reference group for age‐stratified analysis is other atypical antipsychotics with similar age distribution.

Tables 4 and 5 show the association between the use of antipsychotic agents and the secondary outcome of non‐hip/femur fractures for all and treatment‐naïve individuals respectively. For risperidone compared with atypical antipsychotics, there were no statistically significant differences. For typical antipsychotics compared with other atypical antipsychotics, there were no statistically significant differences in the aggregated analysis, but the age‐stratified analysis showed moderately increased risk in the 65+ age group. This was the case for both all users (aHR: 1.19; 95% CI: 1.01–1.40) and those who were treatment‐naïve (aHR: 1.19; 95% CI: 1.00–1.41).

**Table 4:** Secondary outcome (non‐hip/femur fractures). Hazard ratios (HR) and 95% confidence intervals (CI) for the association between the use of risperidone, other atypical antipsychotics, and typical antipsychotics and the secondary outcome, overall and stratified by three age groups using a time on drug analysis

Characteristics	Person‐years of follow‐up	Number of events	Events per 100 000 person‐years	Unadjusted HR (95% CI)	Adjusted HR (95% CI)*
Other atypical antipsychotics	82 882	5236	6317.4	Reference	Reference
Risperidone	44 315	5637	12720.3	1.81 (1.71–1.91)	0.95 (0.89–1.03)
18–44†	11 413	418	3662.5	0.89 (0.76–1.04)	0.88 (0.75–1.04)
45–64	11 941	511	4279.4	1.00 (0.88–1.14)	0.94 (0.82–1.08)
65+	20 962	4708	22459.7	1.10 (1.01–1.21)	0.99 (0.89–1.10)
Typical antipsychotics	26 054	2353	9031.2	1.72 (1.60–1.84)	1.03 (0.95–1.12)
18–44	5274	184	3488.8	1.08 (0.88–1.31)	1.04 (0.85–1.27)
45–64	12 794	510	3986.2	1.02 (0.90–1.17)	0.98 (0.85–1.13)
65+	7986	1659	20773.9	1.18 (1.07–1.31)	1.10 (0.98–1.25)

* Adjusted for age, clinic, multidose dispensing, Charlson index, history of psychiatric in‐patient care, dementia, and stress‐related or somatoform disorder.

^†^ Reference group for age‐stratified analysis is other atypical antipsychotics with similar age distribution.

**Table 5:** Secondary outcome (non‐hip/femur fractures), restricted to treatment‐naïve patients. Hazard ratios (HR) and 95% confidence intervals (CI) for the association between the use of risperidone, other atypical antipsychotics, and typical antipsychotics and the secondary outcome, overall and stratified by three age groups using a time on drug analysis

Characteristics	Person‐years of follow‐up	Number of events	Events per 100 000 person‐years	Unadjusted HR (95% CI)	Adjusted HR (95% CI)*
Other atypical antipsychotics	64 708	4477	6918.8		
Risperidone	37 809	5150	13621.1	1.88 (1.71–2.06)	0.99 (0.88–1.11)
18–44†	9439	334	3538.5	0.89 (0.67–1.18)	0.92 (0.69–1.24)
45–64	9470	408	4308.3	1.01 (0.81–1.27)	0.91 (0.72–1.15)
65+	18 900	4408	23322.8	1.07 (0.94–1.22)	1.03 (0.88–1.20)
Typical antipsychotics	24 557	2245	9142	1.69 (1.51–1.89)	1.06 (0.94–1.21)
18–44	5053	177	3502.9	0.70 (0.46–1.06)	0.72 (0.47–1.10)
45–64	12 006	461	3839.7	1.07 (0.87–1.32)	1.04 (0.84–1.30)
65+	7498	1607	21432.4	1.21 (1.04–1.41)	1.19 (1.00–1.41)

* Adjusted for age, clinic, multidose dispensing, Charlson index, history of psychiatric in‐patient care, dementia, and stress‐related or somatoform disorder.

^†^ Reference group for age‐stratified analysis is other atypical antipsychotics with similar age distribution.

The results were not materially changed in the sensitivity analyses (see the Supporting information).

## Discussion

This study indicates that compared with other atypical antipsychotic agents, risperidone use is not associated with an elevated risk of osteoporosis‐related fractures. For typical antipsychotics, a moderately elevated risk of hip fractures was noted compared with other atypical antipsychotics even after age stratification, but for non‐hip fractures the increased risk was present only in the 65+ age group.

The use of any atypical antipsychotic compared with non‐use has in some studies been associated with a higher risk of fracture 19, whereas in another study, it found no statistically significant associations 20. The results of this study are consistent with the findings of a recent review and meta‐analysis that included 19 observational studies on the association between the use of antipsychotics and fractures where most of the studies considered hip fractures among the elderly 8. The meta‐analysis included studies covering six antipsychotics (chlorpromazine, haloperidol, olanzapine, quetiapine, risperidone, and thioridazine), and risperidone was associated with the lowest fracture risk, with small differences between atypical antipsychotics. Further, a recently published Danish population‐based cohort study of patients aged 65 years or more did not find any significant differences in risk between the atypical antipsychotics (risperidone, olanzapine, and quetiapine) included in the study 10. Moreover, another Danish population‐based cohort study on the risk of hip fracture among patients with schizophrenia did not find an association with any specific antipsychotic drug 9.

Osteoporosis is associated with several risk factors, including age, female sex, lifestyle, certain diseases, and medications. The risk factors act via various mechanisms to affect risks of osteoporosis and of osteoporosis‐related fractures. For example, hyperprolactinemia caused by antipsychotics is believed to reduce bone mineral density and may thus contribute to osteoporosis via a direct and an indirect mechanism: directly by reducing osteoblast cell numbers and indirectly via the hypothalamic–pituitary–gonadal axis by decreasing the levels of gonadal hormones 7. Given the multicausal nature of osteoporosis‐related fractures, it is challenging to disentangle the specific risk contribution of a prolactin‐elevating antipsychotic such as risperidone. We therefore examined a number of potential confounding factors and included in adjusted models those that met a predetermined change‐in‐estimate criterion. There was a moderately increased fracture risk of typical antipsychotics but not risperidone compared with other atypical antipsychotics, and since risperidone is known for its potential to increase prolactin, the increased risk associated with typical antipsychotics is unlikely to have been caused by hyperprolactinemia but perhaps more likely residual confounding related to lifestyle factors that could not be controlled. For instance, schizophrenia is more common in this group and is associated with a sedentary lifestyle 21, 22 and smoking 23. As those data not are available in the present databases, we were unable to fully control for these risk factors for osteoporosis.

Our results further indicate that the higher age and greater frailty among patients who are exposed to risperidone should be carefully considered when investigating associations between fracture and antipsychotic treatment. Higher age is known to be an important risk factor for osteoporosis‐related fractures 24. Multidose dispensing was one of the variables that fulfilled the criteria of a minimum of 10% change in the unadjusted estimate after introducing the candidate covariate in the model. This variable may be a proxy for frailty, because multidose dispensing is commonly used for patients with cognitive disabilities or with physical challenges in handling their medications, and also for patients prescribed a various number of tablets for chronic diseases. In nursing homes in Sweden, it is not uncommon that elderly are prescribed antipsychotics 25. In Sweden, risperidone is approved for short‐term treatment of persistent aggression in patients with moderate‐to‐severe Alzheimer's disease, who do not respond to treatment with non‐pharmacological approaches 26. This practice of antipsychotic treatment in patients with dementia is likely the single most important reason why risperidone users had a higher average age in this study, compared with the other antipsychotic exposure groups.

### Strengths and limitations

The strengths of this study include the use of a population‐based cohort design, including all individuals initiating antipsychotic treatment during the study period and minimal loss to follow‐up. The national health registers in Sweden are known to be of high quality; the recorded psychiatric diagnoses in NPR have a high concordance with the corresponding diagnoses in the Diagnostic and Statistical Manual of Mental Disorders (DSM‐IV) 14, and the PDR provides complete and reliable data 13. The large number of included patients and person‐time antipsychotic exposure generated enough statistical power to detect even a small change in the risk of a fracture.

The descriptive results of this study apply to an in‐patient and out‐patient population who have filled prescriptions of antipsychotics and are new users of antipsychotics in Sweden, except for patients who only received medication during a hospitalization. In our assessment, the study has a design suitable to evaluate the risk of fracture among Swedish individuals who were dispensed antipsychotics, and is generalizable to Swedish patients.

A limitation of this study is that the PDR only provides information on dispensing and not medication use and discontinuation dates. Therefore, person‐time and durations of treatment may to some extent be misclassified. Out‐patient data were not included in the NPR until 2001 and the recording of psychiatric out‐patient diagnoses has been incomplete before 2008 14, which may have led to misclassification of psychiatric diagnosis before the index dispensing. Further, we had no information on diagnoses given by general practitioners in primary care, although care guidelines generally indicate that patients with a severe psychiatric condition should be referred to specialized care 27. Finally, as the PDR does not include data on the indication of treatment, we could not control for this potentially important factor.

Although the study design attempted to limit prior antipsychotic exposure by applying a 12‐month period washout prior to the index date, it is possible that some patients were exposed to antipsychotics before 2005 and that true cumulative exposure to antipsychotics therefore could not be measured in all patients. We have attempted to control for confounding through study design and by adjusting for several factors, but residual confounding cannot be excluded. Confounding by contraindication, in the case when an antipsychotic with sedative properties is not prescribed to a fall‐prone and fragile patient, may be partially avoided by excluding patients with a prior osteoporosis‐related fracture. Smoking could only be partially controlled for using available data (e.g., nicotine use disorder). Our data further cover a significant time span. However, this study would not be able to assess associations between antipsychotics and fractures if the effect to induce osteoporosis was due to prolonged exposure spanning decades.

This study considered osteoporosis‐related fractures for the entire population treated with antipsychotic medication. Further research may consider specific subcategories, including individuals with psychiatric disorders such as schizophrenia or bipolar disorder. The diagnostic category of neurotic and stress‐related disorders was found to be a significant confounder and included in adjusted analyses. Further research is required to uncover the underlying causal mechanism. It may be that the diagnostic category is a proxy for lifestyle factors associated with an increased risk of developing osteoporosis, possibly smoking and sedentary behavior, which we were unable to control for. Similarly, a recent study of elderly initiating antidepressant treatment 28 found a higher risk of fracture both before and after initiating treatment, which was among other things attributed to confounding that could not be controlled for as these measures were not included in available registers.

In conclusion, our results suggest that risperidone use is not associated with an elevated risk of osteoporosis‐related fracture compared with other atypical antipsychotic agents. Results were similar for both hip and non‐hip fractures. For typical antipsychotics, a moderately elevated risk of hip fractures was noted compared with other atypical antipsychotics, possibly because of residual confounding.

## Declaration of interest

The study was a voluntary Post‐Authorisation Safety Study (PASS) funded by Janssen Research and Development, a company that manufactures and sells antipsychotics and conducts research in schizophrenia. Janssen Research and Development did not participate in the data acquisition and analysis. DR and HQ are employees of Janssen Research and Development. RB was supported by a Swedish Research Council Grant 2016‐02362. The other authors declare no personal conflict of interest.

Statistical analyses were performed by TS at the Centre for Pharmacoepidemiology (CPE), Department of Medicine Solna, Karolinska Institutet, Sweden (e‐mail address: tobias.svensson@ki.se).

## Author contributions

All authors took part in the design of the study and the interpretation of the results. TS managed the data analyses. EC and JR managed the literature searches and wrote the first draft of the manuscript. All authors critically revised, contributed to, and have approved the final manuscript.

## Data availability statement

The data that support the findings of this study are available from the Swedish National Board of Health and Welfare (Prescribed Drug Register, National Patient Register, Cause of Death Register, and Swedish Cancer Register) as well as Statistics Sweden (Register of Population and Population Changes). Restrictions apply to the availability of these data, which were used under license for this study.

## Supporting information


**Appendix S1**. Disease codes.
**Appendix S2**. Exposure variables.
**Appendix S3**. Potential confounders.
**Table S1**. Baseline characteristics of patients exposed to risperidone, other atypical antipsychotics and typical antipsychotics.
**Table S2**. Medical history and use of medication prior to the index date.
**Table S3**. Characteristics of new users of risperidone, other atypical antipsychotics and typical antipsychotics.
**Table S4**. Selection of confounding factors based on minimum of 10% change in the crude estimate (overall) hazard ratio (HR) and 95% confidence interval (CI).
**Table S5**. Selection of confounding factors based on minimum of 10% change in the crude estimate hazard ratio (HR) and 95% confidence interval (CI). Includes only individuals up to 44 years old.
**Table S6**. Selection of confounding factors based on minimum of 10% change in the crude estimate hazard ratio (HR) and 95% confidence interval (CI). Includes only individuals between 45 and 64 years old.
**Table S7**. Selection of confounding factors based on minimum of 10% change in the crude estimate hazard ratio (HR) and 95% confidence interval (CI). Includes only individuals 65 years or older.
**Table S8**. Primary outcome (non‐open hip/femur fractures).
**Table S9**. Primary outcome (non‐open hip/femur fractures), restricted to treatment naïve patients.
**Table S10**. Secondary outcome (non‐hip/femur fractures).
**Table S11**. Secondary outcome (non‐hip/femur fractures), restricted to treatment naïve patients.
**Table S12**. Non‐open, non‐pathological hospitalized hip/femur fractures, defined as hip/femur fractures without an explicit code as open‐wound fractures, and with no evidence for bone metastases or major trauma.
**Table S13**. Hazard ratios (HR) and 95% confidence intervals (CI) for association between use of risperidone, other atypical antipsychotics, typical antipsychotics and secondary outcome by fracture site.
**Table S14**. Hazard ratios (HR) and 95% confidence intervals (CI) for association between use of risperidone, other atypical antipsychotics, typical antipsychotics and primary outcome, time on drug
**Table S15**. Prescription of antipsychotics for all study subjects and only those with history of an ICD‐10 F40‐F48 diagnosis.
