# Supplementary material for: Exposure to risperidone versus other antipsychotics and risk of osteoporosis‐related fractures: a population‐based study
Source: Acta Psychiatr Scand. 2019 Oct 11;141(1):74–83. doi: 10.1111/acps.13101 (PMC6973241; doi:10.1111/acps.13101)
Supplement: Supplementary file 1 — Appendix S1. Disease codes. Appendix S2. Exposure variables. Appendix S3. Potential confounders. Table S1. Baseline characteristics of patients exposed to risperidone, other atypical antipsychotics and typical antipsychotics. Table S2. Medical history and use of medication prior to the index date. Table S3. Characteristics of new users of risperidone, other atypical antipsychotics and typical antipsychotics. Table S4. Selection of confounding factors based on minimum of 10% change in the crude estimate (overall) hazard ratio (HR) and 95% confidence interval (CI). Table S5. Selection of confounding factors based on minimum of 10% change in the crude estimate hazard ratio (HR) and 95% confidence interval (CI). Includes only individuals up to 44 years old. Table S6. Selection of confounding factors based on minimum of 10% change in the crude estimate hazard ratio (HR) and 95% confidence interval (CI). Includes only individuals between 45 and 64 years old. Table S7. Selection of confounding factors based on minimum of 10% change in the crude estimate hazard ratio (HR) and 95% confidence interval (CI). Includes only individuals 65 years or older. Table S8. Primary outcome (non‐open hip/femur fractures). Table S9. Primary outcome (non‐open hip/femur fractures), restricted to treatment naïve patients. Table S10. Secondary outcome (non‐hip/femur fractures). Table S11. Secondary outcome (non‐hip/femur fractures), restricted to treatment naïve patients. Table S12. Non‐open, non‐pathological hospitalized hip/femur fractures, defined as hip/femur fractures without an explicit code as open‐wound fractures, and with no evidence for bone metastases or major trauma. Table S13. Hazard ratios (HR) and 95% confidence intervals (CI) for association between use of risperidone, other atypical antipsychotics, typical antipsychotics and secondary outcome by fracture site. Table S14. Hazard ratios (HR) and 95% confidence intervals (CI) for association between use of risperidone, other atypi [file ACPS-141-74-s001.docx]

Supplemental file

Exposure to risperidone versus other antipsychotics and risk of osteoporosis-related fractures: A population based study

Contents

[1. Disease codes 2](#_Toc534221268)

[2. Exposure variables 4](#_Toc534221269)

[3. Potential confounders 6](#_Toc534221270)

[4. Tables 9](#_Toc534221271)

1. Disease codes

| **Fracture sites** | **ICD-10 codes** | |
| --- | --- | --- |
|  | **Closed/open/not specified** | **Open fractures** |
| **Hip/Femoral neck** | **S72.0-S72.2** | **S72.0xx** **B S72.2xxC** |
| **Hip, pathological** | **M84.4** | **/** |
| **Femoral, shaft/unspecified** | **S72.3-S72.9** | **S72.3xxB  S72.3xxC** |
| **Femur, pathological** | **M80.05, M80.85** | **/** |
|  |  |  |
| **Vertebral** | **M48.4, M48.5, S22.0, S22.1, S32.0** | **S22.0xxB S22.1xxB S32.0xxB** |
| **Vertebra, pathologic** | **M80.08, M80.88** | **/** |
| **Ribs** | **S22.3, S22.4** |  |
| **Clavicle** | **S42.0, S42.9** |  |
| **Pathologic, shoulder** | **M80.01, M80.81** | **/** |
| **Humerus** | **S42.2-S42.4** | **S42.2xxB- S42.4xxB** |
| **Humerus, pathologic** | **M80.02, M80.82** | **/** |
| **Distal radius/ulna** | **S52** | **S52.xxxB S52.xxxC** |
| **Radius/ulna, pathologic** | **M80.03, M80.83** | **/** |
| **Wrist** | **S62.0, S62.9** | **S62.0xxB, S62.9xxB** |
| **Pelvis** | **S32.1-S32.9** | **S32.1xxB- S32.9xxB** |
| **Tibia/Fibula** | **S82.1-S82.4** | **S82.1xxB- S82.4xxB, S82.1xxC- S82.4xxC** |
| **Tibia/Fibula, pathologic** | **M80.06, M80.86** | **/** |
|  |  |  |
| **Fractures (any site)** | **Sx2, M48.4, M48.5** | **/** |
| **Fractures, pathologic** | **M80, M84.3- M84.6** | **/** |

2. Exposure variables

***2.1. ATC-codes***

***2.1.1. Atypical Antipsychotics***

Aripiprazole N05AX12

Clozapine N05AH02

Lurasidone N05AE05

Olanzapine N05AH03

Quetiapine N05AH04

Risperidone N05AX08

Sertindole N05AE03

Ziprasidone N05AE04

***2.1.2. Typical Antipsychotics***

*Phenothiazines*

Chlorpromazine N05AA01*

Levomepromazine N05AA02

Promazine N05AA03*

Fluphenazine N05AB02

Perphenazine N05AB03

Prochlorperazine N05AB04*

Trifluoperazine N05AB06*

Thioridazine N05AC02*

*Butyrophenone derivatives*

Haloperidol N05AD01

Melperone N05AD03

Droperidol N05AD08

*Indole derivatives*

Molindone N05AE02*

*Thioxanthene derivatives*

Flupentixol N05AF01

Chlorprothixene N05AF03

Tiotixene N05AF04*

Zuclopenthixol N05AF05

*Diphenylbutylepiperdine derivatives*

Pimozide N05AG02*

*Other*

Loxapine N05AH01

* Currently not in use in Sweden

Asenapine (N05AH05) and dixyrazine (N05AB01) were not included as they were approved for only part of the study period and have not been used as antipsychotics in Sweden. In addition, asenapine has not been made available in Sweden by its marketing authorisation holder.

3. Potential confounders

***3.1. ICD-codes***

***3.1.1. Psychiatric disorders***

Dementia (ICD-10 F00-F03)

Other organic psychiatric disorder (ICD-10 F04-F09)

Alcohol use disorder (ICD-10 F10)

Other substance use disorder (ICD-10 F11-F19)

Schizophrenia (ICD-10 F20)

Other psychosis (ICD-10 F21-F29)

Bipolar disorder (ICD-10 F30-F31)

Unipolar disorder (ICD-10 F32-F33)

Other mood disorder (ICD-10 F34-F39)

Neurotic stress related or somatoform disorder (ICD-10 F40-F48)

Personality disorder (ICD-10 F60-F69)

Mental retardation and autism (ICD-10 F70-F89)

Suicide attempt (ICD-10 X60–X84, Y10–Y34)

***3.1.3. Other medical conditions associated with hyperprolactinemia***

Acromegaly (ICD-10 E22.0)

Hyperprolactinemia (ICD-10 E22.1)

Hypothyroidism (ICD-10 E03)

Sarcoidosis (ICD-10 D86)

Liver cirrhosis (ICD-10 K74.3-K75.6)

Chronic renal failure (ICD-10 N18)

Cushing’s disease (ICD-10 E24)

Polycystic ovary syndrome (ICD-10 E28.2)

***3.1.4. Other conditions and drugs associated with osteoporosis***

Obesity (ICD-10 E66.0-E66.9; orlistat, ATC A08AB01)

Earlier fracture during adulthood (at least 18 years of age; ICD-10 S02, S12, S22, S32, S42, S52, S62, S72, S82, S92)

Risk factors for secondary osteoporosis (E10.0 - E10.9, E28.3, K70.1 - K70.4, K70.9, K71.1, K71.3 - K71.5, K71.7, K72.1, K72.9, K73.0 - K73.2, K73.8 - K73.9, K74.0 - K74.6, K75.2 - K75.4, K75.8 - K75.9, K76.1 - K76.7)

Rheumatoid arthritis (ICD-10 M05.0 - M05.9, M06.0 - M06.9)

Nicotine use disorder (proxy for smoking, last six months; ICD-10 F17.1 - till F17.9; ATC N07BA)

Disease related to alcohol use disorder (ICD-10 F10.1 - F10.9, I85.0, I85.9, I98.3, K29.2, K70.0-K70.9, K85.2, E24.4, G31.2, G62.1, G72.1, I42.6, or ATC N07BB01, N07BB04, N07BB03)

Treatment with cortisone, lithium or antiepileptics (ATC H02A, H02B, N05AN01, N03A)

Treatment with drugs associated with earlier osteoporosis (bisphosphonates ATC M05; denosumab ATC: M05), strontium, ATC M05, calcium, ATC A12AA), vitamin D, ATC: A11CC; raloxifen [SERM], ATC G03XC01; teriparatid, ATC H05AA; parathyroid hormone, ATC H05AA; thyroxine, ATC H03AA; estrogens, ATC G03C; androgens, ATC G03B)

***3.2. ATC-codes***

***3.2.1. Drugs which can induce hyperprolactinemia***

Antidepressants N06A

Buspirone N05BE01

Alprazolam N05BA12

Metoclopramide A03FA01

Domperidone A03FA03

Cisapride A03FA02

Methyldopa C02AB

Reserpine C02AA52

Verapamil C08DA01

Morphine N02AA01

Cimetidine A02BA01

Ranitidine A02BA02

***3.2.4. Drugs used for the treatment of hyperprolactinemia***

Cabergoline G02CB03

Bromocriptine N04BC01

Quinagolide G02CB04

4. Tables

**Table 1. Baseline characteristics of patients exposed to risperidone, other atypical antipsychotics and typical antipsychotics**

|  | **Risperidone** | | **Other atypical** | | **Typical** | |
| --- | --- | --- | --- | --- | --- | --- |
| **Characteristics** | Number | Percent | Number | Percent | Number | Percent |
| Total number included | 38211 |  | 60691 |  | 17445 |  |
| *Gender, N (%)* |  |  |  |  |  |  |
| Male | 15672 | 41.0 | 28120 | 46.3 | 7102 | 40.7 |
| Female | 22539 | 59.0 | 32571 | 53.7 | 10343 | 59.3 |
| Age at inclusion |  |  |  |  |  |  |
| Mean (SD) | 67.9 | 21.5 | 44.4 | 17.4 | 63.3 | 19.1 |
| *Age group, N (%)* |  |  |  |  |  |  |
| 18-44 | 7202 | 18.8 | 32579 | 53.7 | 3217 | 18.4 |
| 45-54 | 2919 | 7.6 | 11456 | 18.9 | 2621 | 15.0 |
| 55-64 | 3144 | 8.2 | 8028 | 13.2 | 2928 | 16.8 |
| 65-74 | 4357 | 11.4 | 4642 | 7.6 | 2635 | 15.1 |
| 75+ | 20589 | 53.9 | 3986 | 6.6 | 6044 | 34.6 |
| *Index year, N (%)* |  |  |  |  |  |  |
| 2006 | 3399 | 8.9 | 3247 | 5.4 | 1515 | 8.7 |
| 2007 | 7463 | 19.5 | 7903 | 13.0 | 3843 | 22.0 |
| 2008 | 6101 | 16.0 | 7148 | 11.8 | 3149 | 18.1 |
| 2009 | 4421 | 11.6 | 7752 | 12.8 | 2327 | 13.3 |
| 2010 | 4588 | 12.0 | 8384 | 13.8 | 1969 | 11.3 |
| 2011 | 4071 | 10.7 | 8622 | 14.2 | 1797 | 10.3 |
| 2012 | 4092 | 10.7 | 8415 | 13.9 | 1533 | 8.8 |
| 2013 | 4076 | 10.7 | 9220 | 15.2 | 1312 | 7.5 |

**Table 2. Medical history and use of medication prior to the index date**

|  | **Risperidone** | | **Other atypical** | | **Typical** | |
| --- | --- | --- | --- | --- | --- | --- |
| **Characteristics** | Number | Percent | Number | Percent | Number | Percent |
| *History of psychiatric conditions* |  |  |  |  |  |  |
| Dementia | 7065 | 18.5 | 1210 | 2.0 | 1670 | 9.6 |
| Other organic psychiatric disorders | 3690 | 9.7 | 2172 | 3.6 | 1257 | 7.2 |
| Alcohol use disorder | 2043 | 5.3 | 7659 | 12.6 | 1284 | 7.4 |
| Other substance use disorders | 1592 | 4.2 | 8033 | 13.2 | 1056 | 6.1 |
| Schizophrenia | 1057 | 2.8 | 2709 | 4.5 | 1176 | 6.7 |
| Other psychosis | 3061 | 8.0 | 6955 | 11.5 | 2013 | 11.5 |
| Bipolar disorder | 958 | 2.5 | 5635 | 9.3 | 514 | 2.9 |
| Unipolar disorder | 5760 | 15.1 | 19184 | 31.6 | 2460 | 14.1 |
| Other mood disorders | 742 | 1.9 | 3270 | 5.4 | 419 | 2.4 |
| Neurotic stress related or somatoform disorder | 5169 | 13.5 | 20130 | 33.2 | 2525 | 14.5 |
| Personality disorder | 1350 | 3.5 | 6195 | 10.2 | 884 | 5.1 |
| Mental retardation and autism | 1116 | 2.9 | 2012 | 3.3 | 401 | 2.3 |
| Suicide attempt | 1766 | 4.6 | 8099 | 13.3 | 940 | 5.4 |
| *Psychiatric inpatient care within 180 days* |  |  |  |  |  |  |
| Mean number of days (SD) | 36.9 | 39.9 | 34.1 | 38.6 | 41.3 | 45.7 |
| None | 31790 |  | 37548 | . | 14446 |  |
| 1-3 | 608 | 1.6 | 2433 | 4.0 | 301 | 1.7 |
| 4-21 | 2349 | 6.1 | 8992 | 14.8 | 1014 | 5.8 |
| 22+ | 3464 | 9.1 | 11718 | 19.3 | 1684 | 9.7 |
| *Psychiatric inpatient care within 5 years* |  |  |  |  |  |  |
| Mean number of days (SD) | 73 | 171.2 | 73 | 177.8 | 107.1 | 257.7 |
| None | 28966 | . | 28331 | . | 12639 | . |
| 1-3 | 933 | 2.4 | 3280 | 5.4 | 503 | 2.9 |
| 4-21 | 2866 | 7.5 | 10287 | 16.9 | 1399 | 8 |
| 22+ | 5446 | 14.3 | 18793 | 31.0 | 2904 | 16.6 |
| *Somatic inpatient care within 180 days* |  |  |  |  |  |  |
| Mean number of days (SD) | 14.9 | 19.9 | 9.4 | 20.5 | 16.3 | 23.2 |
| None | 28657 |  | 50742 |  | 13559 |  |
| 1-3 | 2624 | 6.9 | 5674 | 9.3 | 1085 | 6.2 |
| 4-21 | 4947 | 12.9 | 3165 | 5.2 | 1906 | 10.9 |
| 22+ | 1983 | 5.2 | 1110 | 1.8 | 895 | 5.1 |
| *Somatic inpatient care within 5 years* |  |  |  |  |  |  |
| Mean number of days (SD) | 22.8 | 58.1 | 14.7 | 72.3 | 25.2 | 88.1 |
| None | 15332 |  | 32926 |  | 8093 |  |
| 1-3 | 5307 | 13.9 | 13146 | 21.7 | 2497 | 14.3 |
| 4-21 | 10134 | 26.5 | 10630 | 17.5 | 3999 | 22.9 |
| 22+ | 7438 | 19.5 | 3989 | 6.6 | 2856 | 16.4 |
| *Inpatient and outpatient diagnosis within 180 days* |  |  |  |  |  |  |
| Acromegaly | 0 | 0 | 0 | 0 | 1 | 0 |
| Hyperprolactinemia | 2 | 0 | 8 | 0 | 3 | 0 |
| Hypothyroidism | 422 | 1.1 | 329 | 0.5 | 162 | 0.9 |
| Sarcoidosis | 9 | 0 | 6 | 0 | 4 | 0 |
| Liver cirrhosis | 12 | 0 | 11 | 0 | 11 | 0.1 |
| Chronic renal failure | 131 | 0.3 | 77 | 0.1 | 66 | 0.4 |
| Cushing's disease | 1 | 0 | 0 | 0 | 0 | 0 |
| Polycystic ovary syndrome | 2 | 0 | 26 | 0 | 4 | 0 |
| *Clinic of the prescriber of the index exposure* |  |  |  |  |  |  |
| No information | 32 | 0.1 | 87 | 0.1 | 26 | 0.1 |
| Primary care | 17772 | 46.5 | 3449 | 5.7 | 6660 | 38.2 |
| Other | 2806 | 7.3 | 2716 | 4.5 | 1225 | 7 |
| Somatic clinic | 4403 | 11.5 | 2490 | 4.1 | 1801 | 10.3 |
| Psychiatric clinic | 13198 | 34.5 | 51949 | 85.6 | 7733 | 44.3 |
| *Type of administration of the new drug* |  |  |  |  |  |  |
| Oral | 37180 | 97.3 | 60615 | 99.9 | 15216 | 87.2 |
| Long acting injection | 1031 | 2.7 | 76 | 0.1 | 2229 | 12.8 |
| *Type of dispensing of the index exposure* |  |  |  |  |  |  |
| Multi-dose drug dispensing^1^ | 11899 | 31.1 | 6978 | 11.5 | 3331 | 19.1 |
| Package | 26312 | 68.9 | 53713 | 88.5 | 14114 | 80.9 |
| *Other prolactin-elevating drugs* |  |  |  |  |  |  |
| Buspirone | 224 | 0.6 | 741 | 1.2 | 158 | 0.9 |
| Alprazolam | 990 | 2.6 | 3374 | 5.6 | 577 | 3.3 |
| Morphine | 607 | 1.6 | 353 | 0.6 | 287 | 1.6 |
| Antidepressants | 17526 | 45.9 | 33646 | 55.4 | 6630 | 38.0 |
| Metoclopramide | 342 | 0.9 | 686 | 1.1 | 284 | 1.6 |
| Cisapride | 3 | 0 | 4 | 0 | 3 | 0 |
| Domperidone | 1 | 0 | 39 | 0.1 | 4 | 0 |
| Reserpine | 1 | 0 | 39 | 0.1 | 4 | 0 |
| Verapamil | 236 | 0.6 | 100 | 0.2 | 93 | 0.5 |
| Cimetidine | 3 | 0 | 6 | 0 | 5 | 0 |
| Ranitidine | 308 | 0.8 | 412 | 0.7 | 217 | 1.2 |
| Methyldopa | 0 | 0 | 1 | 0 | 0 | 0 |
| *Drugs used for the treatment of hyperprolactinemia* |  |  |  |  |  |  |
| Bromocriptine | 1 | 0 | 7 | 0 | 1 | 0 |
| Cabergoline | 4 | 0 | 8 | 0 | 1 | 0 |
| Quinagolide | 2 | 0 | 3 | 0 | 2 | 0 |

^1^ Multi-dose dispensing refers to a situation where medication is delivered in separate bags or trays for each individual administration time instead of separate pharmaceutical packaging for each drug.

|  | **Risperidone** | | **Other atypical** | | **Typical** | |
| --- | --- | --- | --- | --- | --- | --- |
| **Characteristics** | Number | Percent | Number | Percent | Number | Percent |
| *Risk factors for osteoporotic fractures based on FRAX* |  |  |  |  |  |  |
| Obesity | 785 | 2.1 | 2810 | 4.6 | 624 | 3.6 |
| Earlier fracture during adulthood | 7711 | 20.2 | 8774 | 14.5 | 3109 | 17.8 |
| Risk factors for secondary osteoporosis | 4440 | 11.6 | 4379 | 7.2 | 2276 | 13 |
| Rheumatoid arthritis | 503 | 1.3 | 425 | 0.7 | 203 | 1.2 |
| Nicotine use disorder | 187 | 0.5 | 473 | 0.8 | 125 | 0.7 |
| Disease related to alcohol use disorder | 2598 | 6.8 | 10466 | 17.2 | 1682 | 9.6 |
| Cortisone | 4511 | 11.8 | 7821 | 12.9 | 2163 | 12.4 |
| Antiepileptics and lithium | 5826 | 15.2 | 22053 | 36.3 | 3086 | 17.7 |

**Table 3. Characteristics of new users of risperidone, other atypical antipsychotics**

**and typical antipsychotics**

|  | **Risperidone** | | **Other atypical** | | **Typical** | |
| --- | --- | --- | --- | --- | --- | --- |
| **Characteristics** | Number | Percent | Number | Percent | Number | Percent |
| Total number included | 38211 |  | 60691 |  | 17445 |  |
| *Person Year total cohort follow-up* |  |  |  |  |  |  |
| Total | 148643 |  | 264423 |  | 79969 |  |
| Mean (SD) | 3.89 | 2.2 | 4.36 | 2.1 | 4.58 | 2.2 |
| *Person Year active treatment* |  |  |  |  |  |  |
| Total | 72192 |  | 112353 |  | 37379 |  |
| Mean (SD) | 1.89 | 1.9 | 1.85 | 2.0 | 2.14 | 2.2 |
| *Number of censored* |  |  |  |  |  |  |
| End of follow-up | 21835 |  | 55460 |  | 12172 |  |
| Emigration | 155 |  | 524 |  | 83 |  |
| Death | 16221 |  | 4707 |  | 5190 |  |
| *Number of osteoporosis-related fracture cases* |  |  |  |  |  |  |
| Primary outcome | 1269 |  | 420 |  | 443 |  |
| Secondary outcome | 5637 |  | 5236 |  | 2353 |  |

**Table 4. Selection of confounding factors based on minimum of 10% change in the crude estimate (overall) hazard ratio (HR) and 95% confidence interval (CI)**

|  | | **Risperidone** | | **Typical AP** | |
| --- | --- | --- | --- | --- | --- |
| **Characteristics** | Other atypical AP | N/Rate/HR and 95% CI | Percent change vs. other atypical | N/Rate/HR and 95% CI | Percent change vs. other atypical |
| Person-years of follow-up | 262873 | 144279 |  | 78199 |  |
| Number of events | 798 | 2753 |  | 1000 |  |
| Events/100,000 person-years | 303.6 | 1908.1 |  | 1278.8 |  |
| Crude |  | 6.27 (5.79-6.78) | 0 | 4.27 (3.89-4.68) | 0 |
| HR adjusted for Age |  | 1.35 (1.24-1.47) | **78.4** | 1.25 (1.14-1.38) | **70.7** |
| HR adjusted for sex |  | 6.12 (5.65-6.62) | 2.4 | 4.16 (3.79-4.57) | 2.5 |
| HR adjusted for clinic of the first dispensation |  | 2.68 (2.46-2.93) | **57.2** | 2.17 (1.95-2.41) | **49.2** |
| HR adjusted for oral/depot |  | 6.46 (5.97-6.99) | 3.2 | 4.65 (4.23-5.11) | 8.9 |
| HR adjusted for multi-dose dispensing |  | 5.58 (5.15-6.04) | **11.0** | 4.06 (3.70-4.46) | 4.8 |
| HR adjusted for somatic inpatient care, 180 days prior |  | 5.94 (5.49-6.43) | 5.2 | 4.08 (3.72-4.48) | 4.4 |
| HR adjusted for somatic inpatient care, 5 years prior |  | 5.85 (5.40-6.33) | 6.7 | 4.09 (3.73-4.49) | 4.1 |
| HR adjusted for psychiatric inpatient care, 180 days prior |  | 5.65 (5.22-6.12) | 9.9 | 4.09 (3.72-4.50) | 4.0 |
| HR adjusted for psychiatric inpatient care, 5 years prior |  | 5.25 (4.84-5.69) | **16.2** | 3.83 (3.48-4.22) | **10.2** |
| *History of psychiatric conditions* |  |  |  |  |  |
| HR adjusted for dementia |  | 5.24 (4.83-5.69) | **16.4** | 3.59 (3.26-3.95) | **15.8** |
| HR adjusted for other organic psychiatric disorders |  | 5.95 (5.49-6.44) | 5.1 | 4.08 (3.71-4.48) | 4.4 |
| HR adjusted for alcohol use disorder |  | 6.08 (5.62-6.58) | 2.9 | 4.19 (3.82-4.60) | 1.7 |
| HR adjusted for other substance use disorder |  | 5.92 (5.47-6.41) | 5.5 | 4.12 (3.75-4.52) | 3.5 |
| HR adjusted for schizophrenia |  | 6.21 (5.74-6.72) | 0.9 | 4.30 (3.92-4.72) | 0.8 |
| HR adjusted for other psychosis |  | 6.20 (5.73-6.71) | 1.0 | 4.27 (3.89-4.68) | 0.0 |
| HR adjusted for bipolar disorder |  | 6.10 (5.64-6.61) | 2.6 | 4.20 (3.83-4.62) | 1.5 |
| HR adjusted for unipolar disorder |  | 5.94 (5.48-6.43) | 5.2 | 4.12 (3.75-4.53) | 3.5 |
| HR adjusted for other mood disorders |  | 6.18 (5.71-6.69) | 1.4 | 4.22 (3.84-4.63) | 1.1 |
| HR adjusted for neurotic stress-related or somatoform disorder |  | 5.62 (5.19-6.09) | **10.3** | 3.91 (3.56-4.30) | 8.3 |
| HR adjusted for personality disorder |  | 5.99 (5.53-6.48) | 4.4 | 4.14 (3.77-4.54) | 3.0 |
| HR adjusted for mental retardation and autism |  | 6.27 (5.8-6.79) | 0.1 | 4.24 (3.86-4.65) | 0.6 |
| HR adjusted for suicide attempt |  | 5.99 (5.54-6.49) | 4.3 | 4.11 (3.74-4.51) | 3.7 |
| *Inpatient and outpatient diagnosis within 180 days prior to the index exposure* |  |  |  |  |  |
| HR adjusted for Acromegaly |  | 6.27 (5.79-6.78) | 0 | 4.27 (3.89-4.68) | 0 |
| HR adjusted for Hyperprolactinemia |  | 6.27 (5.79-6.78) | 0 | 4.27 (3.89-4.68) | 0 |
| HR adjusted for Hypothyroidism |  | 6.23 (5.76-6.75) | 0.5 | 4.25 (3.87-4.66) | 0.4 |
| HR adjusted for Sarcoidosis |  | 6.27 (5.79-6.78) | 0 | 4.27 (3.89-4.69) | 0 |
| HR adjusted for Liver cirrhosis |  | 6.27 (5.79-6.78) | 0 | 4.27 (3.89-4.69) | 0 |
| HR adjusted for Chronic renal failure |  | 6.26 (5.78-6.77) | 0.1 | 4.26 (3.88-4.67) | 0.3 |
| HR adjusted for Cushing’s disease |  | 6.27 (5.79-6.78) | 0 | 4.27 (3.89-4.68) | 0 |
| HR adjusted for Polycystic ovary syndrome |  | 6.26 (5.79-6.78) | 0 | 4.27 (3.89-4.68) | 0 |
| *Other prolactin-elevating drugs* |  |  |  |  |  |
| HR adjusted for Buspirone |  | 6.25 (5.77-6.76) | 0.3 | 4.26 (3.89-4.68) | 0.1 |
| HR adjusted for Alprazolam |  | 6.21 (5.74-6.72) | 0.9 | 4.24 (3.86-4.65) | 0.7 |
| HR adjusted for Morphine |  | 6.24 (5.77-6.76) | 0.4 | 4.21 (3.83-4.62) | 1.4 |
| HR adjusted for antidepressants |  | 6.10 (5.64-6.60) | 2.6 | 4.18 (3.81-4.6) | 1.9 |
| HR adjusted for Metoclopramide |  | 6.27 (5.79-6.78) | 0 | 4.25 (3.88-4.67) | 0.3 |
| HR adjusted for Cisapride |  | 6.27 (5.79-6.78) | 0 | 4.27 (3.89-4.68) | 0 |
| HR adjusted for Domperidone |  | 6.28 (5.80-6.79) | 0.2 | 4.27 (3.89-4.69) | 0.1 |
| HR adjusted for Reserpine |  | 6.27 (5.79-6.78) | 0 | 4.27 (3.89-4.68) | 0 |
| HR adjusted for Verapamil |  | 6.24 (5.77-6.75) | 0.4 | 4.26 (3.88-4.68) | 0.2 |
| HR adjusted for Cimetidine |  | 6.27 (5.79-6.78) | 0 | 4.27 (3.89-4.68) | 0 |
| HR adjusted for Ranitidine |  | 6.27 (5.79-6.78) | 0 | 4.28 (3.90-4.70) | 0.3 |
| HR adjusted for Methyldopa |  | 6.27 (5.79-6.78) | 0 | 4.27 (3.89-4.68) | 0 |
| *Drugs used for the treatment of hyperprolactinemia* |  |  |  |  |  |
| HR adjusted for Bromocriptine |  | 6.27 (5.79-6.78) | 0 | 4.27 (3.89-4.69) | 0.1 |
| HR adjusted for Cabergoline |  | 6.27 (5.79-6.78) | 0 | 4.27 (3.89-4.68) | 0 |
| HR adjusted for Quinagolide |  | 6.27 (5.79-6.78) | 0 | 4.27 (3.89-4.68) | 0 |

|  | | **Risperidone** | | **Typical AP** | |
| --- | --- | --- | --- | --- | --- |
| **Characteristics** | Other atypical AP | N/Rate/HR and 95% CI | Percent change vs. other atypical | N/Rate/HR and 95% CI | Percent change vs. other atypical |
| *Risk factors for osteoporotic fractures based on FRAX* |  |  |  |  |  |
| HR adjusted for obesity |  | 6.27 (5.79-6.78) | 0.0 | 4.27 (3.89-4.68) | 0.0 |
| HR adjusted for fracture during adulthood |  | 6.08 (5.61-6.57) | 3.0 | 4.17 (3.80-4.57) | 2.4 |
| HR adjusted for risk factors for secondary osteoporosis |  | 6.20 (5.73-6.71) | 1.1 | 4.17 (3.80-4.58) | 2.3 |
| HR adjusted for rheumatoid arthritis |  | 6.26 (5.78-6.77) | 0.2 | 4.25 (3.87-4.67) | 0.4 |
| HR adjusted for nicotine use disorder |  | 6.27 (5.79-6.78) | 0.0 | 4.27 (3.89-4.68) | 0.0 |
| HR adjusted for disease related to alcohol use disorder |  | 5.95 (5.5-6.44) | 5.1 | 4.13 (3.76-4.54) | 3.2 |
| HR adjusted for cortisone |  | 6.28 (5.8-6.79) | 0.1 | 4.27 (3.89-4.69) | 0.1 |
| HR adjusted for antiepileptics and lithium |  | 5.70 (5.26-6.17) | 9.1 | 3.99 (3.63-4.38) | 6.6 |
| *Charlson index* |  |  |  |  |  |
| HR adjusted for Charlson index |  | 4.92 (4.53-5.33) | 21.5 | 3.34 (3.04-3.68) | 21.6 |

**Table 5. Selection of confounding factors based on minimum of 10% change in the crude estimate hazard ratio (HR) and 95% confidence interval (CI). Includes only individuals up to 44 years old**

|  | | **Risperidone** | | **Typical AP** | |
| --- | --- | --- | --- | --- | --- |
| **Characteristics** | Other atypical AP | N/Rate/HR and 95% CI | Percent change vs. other atypical | N/Rate/HR and 95% CI | Percent change vs. other atypical |
| Person-years of follow-up | 143344 | 37365 |  | 17764 | . |
| Number of events | 29 | 8 |  | 6 | . |
| Events/100,000 person-years | 20.2 | 21.4 |  | 33.8 | . |
| Crude |  | 0.98(0.45-2.16) | 0 | 1.52(0.63-3.67) | 0 |
| HR adjusted for Age |  | 0.97(0.44-2.14) | 0.9 | 1.38(0.57-3.37) | 8.9 |
| HR adjusted for sex |  | 0.96(0.44-2.1) | 2.6 | 1.53(0.63-3.7) | 0.8 |
| HR adjusted for clinic of the first dispensation |  | 0.94(0.42-2.07) | 4.7 | 1.57(0.64-3.86) | 3.6 |
| HR adjusted for oral/depot |  | 0.94(0.41-2.14) | 4.7 | 1.46(0.57-3.78) | 3.6 |
| HR adjusted for multi-dose dispensing |  | 0.98(0.45-2.14) | 0.6 | 1.53(0.63-3.71) | 0.9 |
| HR adjusted for somatic inpatient care, 180 days prior |  | 1.02(0.46-2.24) | 3.6 | 1.55(0.64-3.75) | 2.1 |
| HR adjusted for somatic inpatient care, 5 years prior |  | 1.08(0.49-2.37) | 9.7 | 1.61(0.66-3.88) | 5.7 |
| HR adjusted for psychiatric inpatient care, 180 days prior |  | 1.01(0.46-2.22) | 2.9 | 1.63(0.67-3.96) | 7.4 |
| HR adjusted for psychiatric inpatient care, 5 years prior |  | 1.01(0.46-2.22) | 2.7 | 1.61(0.66-3.91) | 5.9 |
| *History of psychiatric conditions* |  |  |  |  |  |
| HR adjusted for dementia |  | 0.98(0.45-2.16) | 0 | 1.52(0.63-3.67) | 0.1 |
| HR adjusted for other organic psychiatric disorders |  | 0.97(0.44-2.13) | 1.1 | 1.52(0.63-3.66) | 0.3 |
| HR adjusted for alcohol use disorder |  | 0.99(0.45-2.17) | 0.6 | 1.54(0.64-3.71) | 1.2 |
| HR adjusted for other substance use disorder |  | 1.03(0.47-2.27) | 5.1 | 1.55(0.64-3.75) | 2.2 |
| HR adjusted for schizophrenia |  | 0.98(0.45-2.14) | 0.8 | 1.5(0.62-3.63) | 1.1 |
| HR adjusted for other psychosis |  | 0.97(0.44-2.12) | 1.8 | 1.49(0.62-3.59) | 2.1 |
| HR adjusted for bipolar disorder |  | 1.00(0.46-2.2) | 1.8 | 1.56(0.64-3.77) | 2.5 |
| HR adjusted for unipolar disorder |  | 1.00(0.45-2.19) | 1.2 | 1.6(0.66-3.88) | 5.3 |
| HR adjusted for other mood disorders |  | 0.97(0.44-2.14) | 1.0 | 1.5(0.62-3.63) | 1.0 |
| HR adjusted for neurotic stress-related or somatoform disorder |  | 1.00(0.46-2.2) | 2.0 | 1.6(0.66-3.87) | 5.1 |
| HR adjusted for personality disorder |  | 1.00(0.46-2.19) | 1.7 | 1.53(0.63-3.7) | 0.8 |
| HR adjusted for mental retardation and autism |  | 0.98(0.45-2.17) | 0.1 | 1.52(0.63-3.68) | 0.3 |
| HR adjusted for suicide attempt |  | 1.01(0.46-2.21) | 2.5 | 1.58(0.65-3.82) | 4 |
| *Inpatient and outpatient diagnosis within 180 days prior to the index exposure* |  |  |  |  |  |
| HR adjusted for Acromegaly |  | 0.98(0.45-2.16) | 0 | 1.52(0.63-3.67) | 0 |
| HR adjusted for Hyperprolactinemia |  | 0.98(0.45-2.16) | 0 | 1.52(0.63-3.67) | 0.1 |
| HR adjusted for Hypothyroidism |  | 0.98(0.45-2.15) | 0.2 | 1.52(0.63-3.67) | 0 |
| HR adjusted for Sarcoidosis |  | 0.98(0.45-2.16) | 0 | 1.52(0.63-3.67) | 0 |
| HR adjusted for Liver cirrhosis |  | 0.98(0.45-2.16) | 0 | 1.52(0.63-3.67) | 0 |
| HR adjusted for Chronic renal failure |  | 0.98(0.45-2.16) | 0 | 1.52(0.63-3.67) | 0 |
| HR adjusted for Cushing’s disease |  | 0.98(0.45-2.16) | 0 | 1.52(0.63-3.67) | 0 |
| HR adjusted for Polycystic ovary syndrome |  | 0.98(0.45-2.16) | 0.1 | 1.52(0.63-3.67) | 0 |
| *Other prolactin-elevating drugs* |  |  |  |  |  |
| HR adjusted for Buspirone |  | 0.98(0.45-2.15) | 0.2 | 1.51(0.63-3.65) | 0.6 |
| HR adjusted for Alprazolam |  | 1.01(0.46-2.22) | 2.8 | 1.52(0.63-3.68) | 0.3 |
| HR adjusted for Morphine |  | 0.98(0.45-2.15) | 0.2 | 1.52(0.63-3.68) | 0.3 |
| HR adjusted for antidepressants |  | 0.97(0.44-2.14) | 1 | 1.47(0.61-3.56) | 3.1 |
| HR adjusted for Metoclopramide |  | 0.99(0.45-2.17) | 0.6 | 1.49(0.62-3.61) | 1.7 |
| HR adjusted for Cisapride |  | 0.98(0.45-2.16) | 0 | 1.52(0.63-3.67) | 0 |
| HR adjusted for Domperidone |  | 0.98(0.45-2.16) | 0 | 1.52(0.63-3.67) | 0 |
| HR adjusted for Reserpine |  | 0.98(0.45-2.16) | 0 | 1.52(0.63-3.67) | 0 |
| HR adjusted for Verapamil |  | 0.98(0.45-2.16) | 0 | 1.52(0.63-3.67) | 0 |
| HR adjusted for Cimetidine |  | 0.98(0.45-2.16) | 0 | 1.52(0.63-3.67) | 0.1 |
| HR adjusted for Ranitidine |  | 0.98(0.45-2.16) | 0 | 1.53(0.63-3.69) | 0.6 |
| HR adjusted for Methyldopa |  | 0.98(0.45-2.16) | 0 | 1.52(0.63-3.67) | 0 |
| *Drugs used for the treatment of hyperprolactinemia* |  |  |  |  |  |
| HR adjusted for Bromocriptine |  | 0.98(0.45-2.16) | 0 | 1.52(0.63-3.67) | 0 |
| HR adjusted for Cabergoline |  | 0.98(0.45-2.16) | 0 | 1.52(0.63-3.67) | 0 |
| HR adjusted for Quinagolide |  | 0.98(0.45-2.16) | 0 | 1.52(0.63-3.67) | 0 |

**Table 6. Selection of confounding factors based on minimum of 10% change in the crude estimate hazard ratio (HR) and 95% confidence interval (CI). Includes only individuals between 45 and 64 years old.**

|  | | **Risperidone** | | **Typical AP** | |
| --- | --- | --- | --- | --- | --- |
| **Characteristics** | Other atypical AP | N/Rate/HR and 95% CI | Percent change vs. other atypical | N/Rate/HR and 95% CI | Percent change vs. other atypical |
| Person-years of follow-up | 88073 | 30054 |  | 29600 |  |
| Number of events | 148 | 93 |  | 83 |  |
| Events/100,000 person-years | 168 | 309.4 |  | 280.4 |  |
| Crude |  | 1.81(1.39-2.34) | 0 | 1.63(1.24-2.13) | 0 |
| HR adjusted for Age |  | 1.58(1.21-2.04) | **12.8** | 1.38(1.06-1.82) | **15.0** |
| HR adjusted for sex |  | 1.82(1.4-2.35) | 0.6 | 1.62(1.24-2.12) | 0.4 |
| HR adjusted for clinic of the first dispensation |  | 1.43(1.09-1.87) | **21.0** | 1.37(1.03-1.83) | **15.7** |
| HR adjusted for oral/depot |  | 1.75(1.34-2.29) | 3.1 | 1.63(1.22-2.18) | 0.2 |
| HR adjusted for multi-dose dispensing |  | 1.73(1.34-2.25) | 4.0 | 1.74(1.33-2.28) | 6.8 |
| HR adjusted for somatic inpatient care, 180 days prior |  | 1.77(1.36-2.29) | 2.0 | 1.67(1.28-2.19) | 2.6 |
| HR adjusted for somatic inpatient care, 5 years prior |  | 1.8(1.39-2.33) | 0.3 | 1.67(1.27-2.18) | 2.3 |
| HR adjusted for psychiatric inpatient care, 180 days prior |  | 1.82(1.4-2.36) | 0.8 | 1.75(1.33-2.31) | 7.5 |
| HR adjusted for psychiatric inpatient care, 5 years prior |  | 1.84(1.42-2.39) | 1.9 | 1.72(1.31-2.27) | 5.9 |
| *History of psychiatric conditions* |  |  |  |  |  |
| HR adjusted for dementia |  | 1.66(1.27-2.17) | 8.1 | 1.61(1.23-2.11) | 1.1 |
| HR adjusted for other organic psychiatric disorders |  | 1.74(1.34-2.26) | 3.5 | 1.63(1.25-2.14) | 0.3 |
| HR adjusted for alcohol use disorder |  | 1.85(1.43-2.4) | 2.6 | 1.67(1.28-2.19) | 2.7 |
| HR adjusted for other substance use disorder |  | 1.81(1.40-2.35) | 0.2 | 1.65(1.26-2.16) | 1.2 |
| HR adjusted for schizophrenia |  | 1.8(1.39-2.34) | 0.3 | 1.61(1.23-2.11) | 1.3 |
| HR adjusted for other psychosis |  | 1.78(1.37-2.31) | 1.3 | 1.62(1.24-2.12) | 0.7 |
| HR adjusted for bipolar disorder |  | 1.83(1.41-2.37) | 1.2 | 1.63(1.25-2.14) | 0.4 |
| HR adjusted for unipolar disorder |  | 1.82(1.40-2.36) | 0.5 | 1.67(1.27-2.20) | 2.5 |
| HR adjusted for other mood disorders |  | 1.81(1.39-2.34) | 0 | 1.63(1.25-2.14) | 0.2 |
| HR adjusted for neurotic stress-related or somatoform disorder |  | 1.76(1.36-2.28) | 2.6 | 1.62(1.23-2.13) | 0.5 |
| HR adjusted for personality disorder |  | 1.82(1.4-2.36) | 0.8 | 1.64(1.25-2.15) | 0.6 |
| HR adjusted for mental retardation and autism |  | 1.80(1.39-2.33) | 0.3 | 1.62(1.24-2.13) | 0.3 |
| HR adjusted for suicide attempt |  | 1.83(1.41-2.38) | 1.6 | 1.66(1.27-2.18) | 2.1 |
| *Inpatient and outpatient diagnosis within 180 days prior to the index exposure* |  |  |  |  |  |
| HR adjusted for Acromegaly |  | 1.81(1.39-2.34) | 0 | 1.63(1.24-2.13) | 0 |
| HR adjusted for Hyperprolactinemia |  | 1.81(1.39-2.34) | 0.1 | 1.63(1.24-2.13) | 0.1 |
| HR adjusted for Hypothyroidism |  | 1.81(1.39-2.34) | 0 | 1.63(1.25-2.14) | 0.2 |
| HR adjusted for Sarcoidosis |  | 1.81(1.39-2.34) | 0 | 1.63(1.24-2.13) | 0 |
| HR adjusted for Liver cirrhosis |  | 1.81(1.39-2.34) | 0.1 | 1.63(1.24-2.13) | 0 |
| HR adjusted for Chronic renal failure |  | 1.81(1.39-2.34) | 0.1 | 1.63(1.24-2.13) | 0 |
| HR adjusted for Cushing’s disease |  | 1.81(1.39-2.34) | 0 | 1.63(1.24-2.13) | 0 |
| HR adjusted for Polycystic ovary syndrome |  | 1.81(1.39-2.34) | 0.1 | 1.63(1.24-2.13) | 0 |
| *Other prolactin-elevating drugs* |  |  |  |  |  |
| HR adjusted for Buspirone |  | 1.81(1.39-2.34) | 0 | 1.63(1.24-2.13) | 0 |
| HR adjusted for Alprazolam |  | 1.79(1.38-2.32) | 1.1 | 1.62(1.24-2.13) | 0.2 |
| HR adjusted for Morphine |  | 1.81(1.39-2.34) | 0.1 | 1.63(1.24-2.13) | 0.2 |
| HR adjusted for antidepressants |  | 1.77(1.36-2.29) | 2.1 | 1.54(1.17-2.02) | 5.7 |
| HR adjusted for Metoclopramide |  | 1.81(1.39-2.35) | 0.1 | 1.62(1.24-2.13) | 0.3 |
| HR adjusted for Cisapride |  | 1.81(1.39-2.34) | 0 | 1.63(1.24-2.13) | 0 |
| HR adjusted for Domperidone |  | 1.82(1.40-2.36) | 0.5 | 1.63(1.25-2.14) | 0.2 |
| HR adjusted for Verapamil |  | 1.8(1.39-2.34) | 0.1 | 1.63(1.24-2.13) | 0 |
| HR adjusted for Cimetidine |  | 1.81(1.39-2.34) | 0.1 | 1.63(1.24-2.13) | 0 |
| HR adjusted for Ranitidine |  | 1.81(1.39-2.34) | 0 | 1.63(1.25-2.14) | 0.3 |
| HR adjusted for Methyldopa |  | 1.81(1.39-2.34) | 0 | 1.63(1.24-2.13) | 0 |
| *Drugs used for the treatment of hyperprolactinemia* |  |  |  |  |  |
| HR adjusted for Bromocriptine |  | 1.80(1.39-2.34) | 0.1 | 1.63(1.24-2.13) | 0.1 |
| HR adjusted for Cabergoline |  | 1.81(1.39-2.34) | 0 | 1.63(1.24-2.13) | 0.1 |
| HR adjusted for Quinagolide |  | 1.81(1.39-2.34) | 0.1 | 1.63(1.24-2.13) | 0 |

**Table 7. Selection of confounding factors based on minimum of 10% change in the crude estimate hazard ratio (HR) and 95% confidence interval (CI). Includes only individuals 65 years or older**

|  | | Risperidone | | Typical AP | |
| --- | --- | --- | --- | --- | --- |
| **Characteristics** | Other atypical AP | N/Rate/HR and 95% CI | Percent change vs. other atypical | N/Rate/HR and 95% CI | Percent change vs. other atypical |
| Person-years of follow-up | 31456 | 76859 |  | 30834 |  |
| Number of events | 621 | 2652 |  | 911 |  |
| Events/100,000 person-years | 1974.2 | 3450.5 |  | 2954.5 |  |
| Crude |  | 1.77(1.62-1.93) | 0 | 1.5(1.36-1.67) | 0 |
| HR adjusted for Age |  | 1.21(1.1-1.33) | **31.7** | 1.12(1.01-1.25) | **25.3** |
| HR adjusted for sex |  | 1.75(1.61-1.91) | 1 | 1.49(1.35-1.65) | 0.8 |
| HR adjusted for clinic of the first dispensation |  | 1.34(1.21-1.48) | **24.4** | 1.23(1.1-1.38) | **18.3** |
| HR adjusted for oral/depot |  | 1.78(1.63-1.94) | 0.5 | 1.55(1.4-1.72) | 3 |
| HR adjusted for multi-dose dispensing |  | 1.76(1.61-1.92) | 0.8 | 1.50(1.36-1.66) | 0.1 |
| HR adjusted for somatic inpatient care, 180 days prior |  | 1.77(1.62-1.93) | 0.1 | 1.51(1.36-1.67) | 0.4 |
| HR adjusted for somatic inpatient care, 5 years prior |  | 1.76(1.61-1.92) | 0.6 | 1.50(1.35-1.66) | 0.4 |
| HR adjusted for psychiatric inpatient care, 180 days prior |  | 1.64(1.50-1.79) | 7.5 | 1.47(1.32-1.63) | 2.2 |
| HR adjusted for psychiatric inpatient care, 5 years prior |  | 1.61(1.47-1.77) | 8.9 | 1.43(1.29-1.59) | 4.7 |
| *History of psychiatric conditions* |  |  |  |  |  |
| HR adjusted for dementia |  | 1.72(1.57-1.87) | 3.1 | 1.46(1.32-1.62) | 2.9 |
| HR adjusted for other organic psychiatric disorders |  | 1.76(1.62-1.93) | 0.3 | 1.51(1.36-1.67) | 0.3 |
| HR adjusted for alcohol use disorder |  | 1.76(1.61-1.92) | 0.5 | 1.50(1.35-1.66) | 0.3 |
| HR adjusted for other substance use disorder |  | 1.75(1.61-1.92) | 0.9 | 1.50(1.36-1.66) | 0.2 |
| HR adjusted for schizophrenia |  | 1.75(1.60-1.91) | 1.4 | 1.50(1.36-1.67) | 0.1 |
| HR adjusted for other psychosis |  | 1.75(1.60-1.91) | 1.1 | 1.50(1.36-1.66) | 0.1 |
| HR adjusted for bipolar disorder |  | 1.71(1.57-1.87) | 3.3 | 1.46(1.32-1.62) | 2.7 |
| HR adjusted for unipolar disorder |  | 1.71(1.57-1.87) | 3.3 | 1.46(1.32-1.62) | 2.7 |
| HR adjusted for other mood disorders |  | 1.76(1.61-1.92) | 0.8 | 1.49(1.34-1.65) | 0.9 |
| HR adjusted for neurotic stress-related or somatoform disorder |  | 1.73(1.58-1.89) | 2.3 | 1.47(1.33-1.63) | 2.1 |
| HR adjusted for personality disorder |  | 1.76(1.61-1.92) | 0.8 | 1.5(1.35-1.66) | 0.3 |
| HR adjusted for mental retardation and autism |  | 1.77(1.62-1.93) | 0.1 | 1.51(1.36-1.67) | 0.1 |
| HR adjusted for suicide attempt |  | 1.75(1.6-1.91) | 1.2 | 1.48(1.34-1.64) | 1.5 |
| *Inpatient and outpatient diagnosis within 180 days prior to the index exposure* |  |  |  |  |  |
| HR adjusted for Acromegaly |  | 1.77(1.62-1.93) | 0 | 1.5(1.36-1.67) | 0 |
| HR adjusted for Hyperprolactinemia |  | 1.77(1.62-1.93) | 0 | 1.50(1.36-1.67) | 0 |
| HR adjusted for Hypothyroidism |  | 1.77(1.62-1.93) | 0.1 | 1.50(1.36-1.67) | 0 |
| HR adjusted for Sarcoidosis |  | 1.77(1.62-1.93) | 0 | 1.50(1.36-1.67) | 0 |
| HR adjusted for Liver cirrhosis |  | 1.77(1.62-1.93) | 0 | 1.50(1.36-1.67) | 0.1 |
| HR adjusted for Chronic renal failure |  | 1.77(1.62-1.93) | 0 | 1.50(1.36-1.66) | 0 |
| HR adjusted for Cushing’s disease |  | 1.77(1.62-1.93) | 0 | 1.50(1.36-1.67) | 0 |
| HR adjusted for Polycystic ovary syndrome |  | 1.77(1.62-1.93) | 0 | 1.50(1.36-1.67) | 0 |
| *Other prolactin-elevating drugs* |  |  |  |  |  |
| HR adjusted for Buspirone |  | 1.77(1.62-1.93) | 0.1 | 1.50(1.36-1.66) | 0.2 |
| HR adjusted for Alprazolam |  | 1.75(1.61-1.92) | 0.9 | 1.48(1.34-1.64) | 1.5 |
| HR adjusted for Morphine |  | 1.77(1.62-1.93) | 0 | 1.50(1.35-1.66) | 0.5 |
| HR adjusted for antidepressants |  | 1.73(1.58-1.89) | 2.4 | 1.49(1.35-1.66) | 0.7 |
| HR adjusted for Metoclopramide |  | 1.77(1.62-1.93) | 0.1 | 1.50(1.36-1.67) | 0 |
| HR adjusted for Cisapride |  | 1.77(1.62-1.93) | 0 | 1.50(1.36-1.66) | 0 |
| HR adjusted for Domperidone |  | 1.77(1.62-1.93) | 0.1 | 1.50(1.36-1.66) | 0.1 |
| HR adjusted for Reserpine |  | 1.77(1.62-1.93) | 0 | 1.50(1.36-1.67) | 0 |
| HR adjusted for Verapamil |  | 1.77(1.62-1.93) | 0.1 | 1.51(1.36-1.67) | 0.2 |
| HR adjusted for Cimetidine |  | 1.77(1.62-1.93) | 0 | 1.50(1.36-1.67) | 0 |
| HR adjusted for Ranitidine |  | 1.77(1.62-1.93) | 0 | 1.51(1.36-1.67) | 0.2 |
| HR adjusted for Methyldopa |  | 1.77(1.62-1.93) | 0 | 1.50(1.36-1.67) | 0 |
| *Drugs used for the treatment of hyperprolactinemia* |  |  |  |  |  |
| HR adjusted for Bromocriptine |  | 1.77(1.62-1.93) | 0.1 | 1.51(1.36-1.67) | 0.1 |
| HR adjusted for Cabergoline |  | 1.77(1.62-1.93) | 0 | 1.50(1.36-1.67) | 0 |
| HR adjusted for Quinagolide |  | 1.77(1.62-1.93) | 0 | 1.50(1.36-1.67) | 0 |

**Table 8. Primary outcome (non-open hip/femur fractures)**

Hazard ratios (HRs) and 95% conﬁdence intervals (CIs) for the association between use of risperidone, other atypical antipsychotics, typical antipsychotics and the primary outcome, overall and stratified by three age groups using an intention to treat analysis.

| **Characteristics** | **Person-years of follow-up** | **Number of events** | **Events per**  **100,000**  **person-years** | **Unadjusted**  **HR (95% CI)** | **Adjusted**  **HR**  **(95% CI)^1^** |
| --- | --- | --- | --- | --- | --- |
| *Other atypical antipsychotics* | 111721 | 798 | 303.6 | Reference | Reference |
| *Risperidone* | 144279 | 2753 | 1908.1 | 6.27  (5.79-6.78) | 1.12  (1.01-1.23) |
| 18-44**^2^** | 37365 | 8 | 21.4 | 0.98  (0.45-2.16) | 0.98  (0.44-2.17) |
| 45-64 | 30054 | 93 | 309.4 | 1.81  (1.39-2.34) | 1.21  (0.91-1.60) |
| 65+ | 76859 | 2652 | 3450.5 | 1.77  (1.62-1.93) | 1.06  (0.96-1.17) |
| *Typical antipsychotics* | 78199 | 1000 | 1278.8 | 4.27  (3.89-4.68) | 1.09  (0.98-1.21) |
| 18-44 | 17764 | 6 | 33.8 | 1.52  (0.63-3.67) | 1.51  (0.61-3.75) |
| 45-64 | 29600 | 83 | 280.4 | 1.63  (1.24-2.13) | 1.36  (1.02-1.82) |
| 65+ | 30834 | 911 | 2954.5 | 1.50  (1.36-1.67) | 1.06  (0.95-1.19) |

**^1^** Adjusted for age, clinic, multi-dose dispensing, Charlson index, history of psychiatric inpatient care, dementia, and stress-related or somatoform disorder.

**^2^** Reference group for age stratified analysis is other atypical antipsychotics with similar age distribution.

**Table 9. Primary outcome (non-open hip/femur fractures), restricted to treatment naïve patients**

Hazard ratios (HR) and 95% conﬁdence intervals (CI) for the association between use of risperidone, other atypical antipsychotics, typical antipsychotics and the primary outcome, overall and stratified by three age groups using an intention to treat analysis.

| **Characteristics** | **Person-years of follow-up** | **Number of events** | **Events per**  **100,000**  **person-years** | **Unadjusted**  **HR (95% CI)** | **Adjusted**  **HR**  **(95% CI)^1^** |
| --- | --- | --- | --- | --- | --- |
| Other atypical antipsychotics | 218739 | 656 | 299.9 | Reference | Reference |
| Risperidone | 126786 | 2568 | 2025.5 | 6.74  (6.19-7.34) | 1.12  (1.01-1.25) |
| 18-44**^2^** | 31191 | 7 | 22.4 | 1.21  (0.51-2.85) | 1.17  (0.48-2.81) |
| 45-64 | 24516 | 71 | 289.6 | 1.89  (1.40-2.55) | 1.12  (0.81-1.56) |
| 65+ | 71079 | 2490 | 3503.1 | 1.78  (1.62-1.95) | 1.07  (0.96-1.20) |
| Typical antipsychotics | 74374 | 977 | 1313.6 | 4.46  (4.04-4.93) | 1.11  (0.99-1.25) |
| 18-44 | 17030 | 6 | 35.2 | 1.88  (0.75-4.67) | 1.86  (0.72-4.79) |
| 45-64 | 27671 | 76 | 274.7 | 1.80  (1.34-2.42) | 1.43  (1.04-1.97) |
| 65+ | 29673 | 895 | 3016.2 | 1.52  (1.36-1.69) | 1.08  (0.96-1.22) |

**^1^** Adjusted for age, clinic, multi-dose dispensing, Charlson index, history of psychiatric inpatient care, dementia, and stress-related or somatoform disorder.

**^2^** Reference group for age stratified analysis is other atypical antipsychotics with similar age distribution.

**Table 10. Secondary outcome (non-hip/femur fractures)**

Hazard ratios (HR) and 95% conﬁdence intervals (CI) for the association between use of risperidone, other atypical antipsychotics, typical antipsychotics and the secondary outcome, overall and stratified by three age groups using an intention to treat analysis.

| **Characteristics** | **Person-years of follow-up** | **Number of events** | **Events per**  **100,000**  **person-years** | **Unadjusted**  **HR (95% CI)** | **Adjusted**  **HR**  **(95% CI)^1^** |
| --- | --- | --- | --- | --- | --- |
| Other atypical antipsychotics | 222090 | 6556 | 2952 | Reference | Reference |
| Risperidone | 110238 | 6772 | 6143.1 | 1.81  (1.71-1.91) | 0.95  (0.89-1.03) |
| 18-44**^2^** | 33435 | 540 | 1615.1 | 0.89  (0.76-1.04) | 0.88  (0.75-1.04) |
| 45-64 | 25913 | 679 | 2620.3 | 1.00  (0.88-1.14) | 0.94  (0.82-1.08) |
| 65+ | 50890 | 5553 | 10911.8 | 1.10  (1.01-1.21) | 0.99  (0.89-1.10) |
| Typical antipsychotics | 63023 | 2961 | 4698.3 | 1.72  (1.6-1.84) | 1.03  (0.95-1.12) |
| 18-44 | 15916 | 269 | 1690.1 | 1.08  (0.88-1.31) | 1.04  (0.85-1.27) |
| 45-64 | 25684 | 660 | 2569.7 | 1.02  (0.9-1.17) | 0.98  (0.85-1.13) |
| 65+ | 21422 | 2032 | 9485.6 | 1.18  (1.07-1.31) | 1.10  (0.98-1.25) |

**^1^** Adjusted for age, clinic, multi-dose dispensing, Charlson index, history of psychiatric inpatient care, dementia, and stress-related or somatoform disorder.

**^2^** Reference group for age stratified analysis is other atypical antipsychotics with similar age distribution.

**Table 11. Secondary outcome (non-hip/femur fractures), restricted to treatment naïve patients**

Hazard ratios (HR) and 95% conﬁdence intervals (CI) for the association between use of risperidone, other atypical antipsychotics, typical antipsychotics and the secondary outcome, overall and stratified by three age groups using an intention to treat analysis.

| **Characteristics** | **Person-years of follow-up** | **Number of events** | **Events per**  **100,000**  **person-years** | **Unadjusted**  **HR (95% CI)** | **Adjusted**  **HR**  **(95% CI)^1^** |
| --- | --- | --- | --- | --- | --- |
| Other atypical antipsychotics | 183779 | 5587 | 3040.1 | Reference | Reference |
| Risperidone | 96061 | 6191 | 6444.9 | 1.90  (1.78-2.02) | 0.97  (0.89-1.05) |
| 18-44**^2^** | 28064 | 438 | 1560.7 | 0.88  (0.74-1.04) | 0.88  (0.74-1.05) |
| 45-64 | 21222 | 542 | 2554 | 1.02  (0.88-1.18) | 0.94  (0.81-1.10) |
| 65+ | 46775 | 5211 | 11140.6 | 1.12  (1.01-1.23) | 1.01  (0.90-1.13) |
| Typical antipsychotics | 59970 | 2831 | 4720.7 | 1.74  (1.62-1.88) | 1.03  (0.94-1.12) |
| 18-44 | 15257 | 254 | 1664.8 | 1.01  (0.82-1.24) | 0.97  (0.78-1.20) |
| 45-64 | 24165 | 602 | 2491.2 | 1.05  (0.91-1.21) | 0.98  (0.84-1.14) |
| 65+ | 20549 | 1975 | 9611.2 | 1.19  (1.07-1.33) | 1.12  (0.98-1.27) |

**^1^** Adjusted for age, clinic, multi-dose dispensing, Charlson index, history of psychiatric inpatient care, dementia, and stress-related or somatoform disorder.

**^2^** Reference group for age stratified analysis is other atypical antipsychotics with similar age distribution.

**Table 12. Non-open, non-pathological hospitalized hip/femur fractures, defined as hip/femur fractures without an explicit code as open-wound fractures, and with no evidence for bone metastases or major trauma**

| **Characteristics** | **Number of events** | **Unadjusted HR (95% CI)** | **Adjusted HR (95% CI)*** |
| --- | --- | --- | --- |
| Other atypicals | 56 | Reference | Reference |
| Risperidone | 111 | 3.05 (2.21-4.21) | 0.85 (0.57-1.26) |
| Typicals | 56 | 2.98 (2.06-4.32) | 1.41 (0.93-2.13) |

* Adjusted for age, clinic, multi-dose dispensing, history of psychiatric inpatient care, dementia, and stress-related or somatoform disorder.

**Table 13. Hazard ratios (HR) and 95% confidence intervals (CI) for association between use of risperidone, other atypical antipsychotics, typical antipsychotics and secondary outcome by fracture site**

| **Characteristics** | **Unadjusted HR (95% CI)** | **Adjusted HR (95% CI)*** |
| --- | --- | --- |
| *Risperidone vs. other atypicals* |  |  |
| Vertebral | 2.05 (1.76-2.39) | 0.89 (0.73-1.09) |
| Clavicle | 1.14 (0.88-1.46) | 0.72 (0.52-0.99) |
| Humerus | 2.04 (1.80-2.32) | 1.09 (0.93-1.28) |
| Radius/ulna | 1.62 (1.47-1.8) | 0.98 (0.86-1.11) |
| Wrist | 0.44 (0.28-0.7) | 0.63 (0.38-1.04) |
| Pelvis | 4.17 (3.46-5.03) | 1.40 (1.10-1.78) |
| Tibia/fibula | 1.09 (0.90-1.32) | 1.10 (0.87-1.37) |
| *Typicals vs. other atypicals* |  |  |
| Vertebral | 2.15 (1.79-2.58) | 1.01 (0.81-1.26) |
| Clavicle | 1.01 (0.73-1.4) | 0.72 (0.49-1.04) |
| Humerus | 2.06 (1.77-2.39) | 1.27 (1.07-1.51) |
| Radius/ulna | 1.54 (1.36-1.74) | 1.01 (0.87-1.16) |
| Wrist | 0.42 (0.23-0.76) | 0.61 (0.32-1.19) |
| Pelvis | 3.07 (2.44-3.85) | 1.25 (0.95-1.66) |
| Tibia/fibula | 1.16 (0.92-1.46) | 1.14 (0.88-1.48) |

* Adjusted for age, clinic, multi-dose dispensing, history of psychiatric inpatient care, dementia, and stress-related or somatoform disorder.

**Table 14. Hazard ratios (HR) and 95% confidence intervals (CI) for association between use of risperidone, other atypical antipsychotics, typical antipsychotics and primary outcome, time on drug. The analysis restricted to individuals with at least 6 months exposure to the respective index antipsychotic**

| **Characteristics** | **Number of events** | **Unadjusted HR (95% CI)** | **Adjusted HR (95% CI)*** |
| --- | --- | --- | --- |
| Other atypicals | 420 | Reference | Reference |
| Risperidone | 1269 | 4.70 (4.21-5.25) | 1.04 (0.91-1.19) |
| Typicals | 443 | 3.17 (2.77-3.62) | 1.24 (1.07-1.45) |

* Adjusted for age, clinic, multi-dose dispensing, history of psychiatric inpatient care, dementia, and stress-related or somatoform disorder.

**Table 15. Prescription of antipsychotics for all study subjects and only those with history of an ICD-10 F40 - F48 diagnosis**

|  | **All study subjects** | | **Only those with a history of ICD-10 F40-F48*** | |
| --- | --- | --- | --- | --- |
|  | **116347 (100.0)** | | **35419 (30.4)** | |
| **ATC** | ***Number of patients*** | ***% patients*** | ***Number of patients*** | ***% patients*** |
| ***N05AA01*** | 108 | 0,1% | 21 | 0.1% |
| ***N05AA02*** | 7063 | 6,1% | 2771 | 7.8% |
| ***N05AB01*** | 125 | 0.1% | 54 | 0.2% |
| ***N05AB02*** | 133 | 0.1% | 6 | 0.0% |
| ***N05AB03*** | 3587 | 3.1% | 705 | 2.0% |
| ***N05AB04*** | 853 | 0.7% | 133 | 0.4% |
| ***N05AC02*** | 123 | 0.1% | 16 | 0.0% |
| ***N05AD01*** | 10734 | 9.2% | 1557 | 4.4% |
| ***N05AD03*** | 655 | 0.6% | 183 | 0.5% |
| ***N05AE03*** | 30 | 0.0% | 7 | 0.0% |
| ***N05AE04*** | 867 | 0.7% | 339 | 1.0% |
| ***N05AF01*** | 5403 | 4.6% | 1685 | 4.8% |
| ***N05AF03*** | 872 | 0.7% | 451 | 1.3% |
| ***N05AF05*** | 4821 | 4.1% | 911 | 2.6% |
| ***N05AG02*** | 42 | 0.0% | 10 | 0.0% |
| ***N05AH02*** | 2318 | 2.0% | 372 | 1.1% |
| ***N05AH03*** | 33684 | 29.0% | 13145 | 37.1% |
| ***N05AH04*** | 29474 | 25.3% | 13914 | 39.3% |
| ***N05AL01*** | 3 | 0.0% | 0 | 0.0% |
| ***N05AL03*** | 1 | 0.0% | 0 | 0.0% |
| ***N05AL05*** | 3 | 0.0% | 0 | 0.0% |
| ***N05AN01*** | 9285 | 8.0% | 2914 | 8.2% |
| ***N05AX08*** | 41305 | 35.5% | 7304 | 20.6% |
| ***N05AX12*** | 12865 | 11.1% | 5191 | 14.7% |
| ***N05AX13*** | 1 | 0.0% | 0 | 0.0% |
| ***N05BA01*** | 15093 | 13.0% | 6124 | 17.3% |
| ***N05BA02*** | 2 | 0.0% | 0 | 0.0% |
| ***N05BA04*** | 35064 | 30.1% | 10264 | 29.0% |
| ***N05BA06*** | 1242 | 1.1% | 450 | 1.3% |
| ***N05BA09*** | 21 | 0.0% | 6 | 0.0% |
| ***N05BA12*** | 6312 | 5.4% | 3362 | 9.5% |
| ***N05BB01*** | 17892 | 15.4% | 8239 | 23.3% |
| ***N05BC01*** | 6 | 0.0% | 2 | 0.0% |
| ***N05BE01*** | 1772 | 1.5% | 1012 | 2.9% |
| ***N05CC01*** | 31 | 0.0% | 3 | 0.0% |
| ***N05CD02*** | 4601 | 4.0% | 1762 | 5.0% |
| ***N05CD03*** | 1745 | 1.5% | 530 | 1.5% |
| ***N05CD05*** | 211 | 0.2% | 100 | 0.3% |
| ***N05CD08*** | 274 | 0.2% | 28 | 0.1% |
| ***N05CF01*** | 40509 | 34.8% | 14272 | 40.3% |
| ***N05CF02*** | 15718 | 13.5% | 6493 | 18.3% |
| ***N05CF03*** | 638 | 0.5% | 326 | 0.9% |
| ***N05CH01*** | 3179 | 2.7% | 1492 | 4.2% |
| ***N05CM02*** | 7806 | 6.7% | 722 | 2.0% |
| ***N05CM05*** | 12 | 0.0% | 1 | 0.0% |
| ***N05CM06*** | 25329 | 21.8% | 10438 | 29.5% |
| ***N05CM09*** | 85 | 0.1% | 32 | 0.1% |
